# Therapeutic targeting of BAP1/ASXL3 sub-complex in ASCL1-dependent small cell lung cancer

**DOI:** 10.1038/s41388-022-02240-x

**Published:** 2022-02-22

**Authors:** Natsumi Tsuboyama, Ru Wang, Aileen Patricia Szczepanski, Huanhuan Chen, Zibo Zhao, Lei Shi, Lu Wang

**Affiliations:** 1 Department of Biochemistry and Molecular Genetics, Feinberg School of Medicine, Northwestern University, Chicago, IL 60611 USA; 2 Simpson Querrey Center for Epigenetics, Feinberg School of Medicine, Northwestern University, Chicago, IL 60611 USA; 3 Jiangsu Key Laboratory of Drug Design and Optimization, Department of Medicinal Chemistry, China Pharmaceutical University, Nanjing, 210009 China

**Keywords:** Small-cell lung cancer, Epigenetics

## Abstract

Small cell lung cancer (SCLC) is an aggressive disease, with patients diagnosed with either early-stage, limited stage, or extensive stage of SCLC tumor progression. Discovering and targeting the functional biomarkers for SCLC will be crucial in understanding the molecular basis underlying SCLC tumorigenesis to better assist in improving clinical treatment. Emerging studies have demonstrated that dysregulations in BAP1 histone H2A deubiquitinase complex are collectively associated with pathogenesis in human SCLC. Here, we investigated the function of the oncogenic BAP1/ASXL3/BRD4 epigenetic axis in SCLC by developing a next-generation BAP1 inhibitor, iBAP-II, and focusing on the epigenetic balance established between BAP1 and non-canonical PRC1 complexes in regulating SCLC-specific transcriptional programming. We further demonstrated that pharmacologic inhibition of BAP1’s catalytic activity disrupted BAP1/ASXL3/BRD4 epigenetic axis by inducing protein degradation of the ASXL3 scaffold protein, which bridges BRD4 and BAP1 at active enhancers. Furthermore, treatment of iBAP-II represses neuroendocrine lineage-specific ASCL1/MYCL/E2F signaling in SCLC cell lines, and dramatically inhibits SCLC cell viability and tumor growth in vivo. In summary, this study has provided mechanistic insight into the oncogenic function of BAP1 in SCLC and highlighted the potential of targeting BAP1’s activity as a novel SCLC therapy.

## Introduction

Lung cancer is one of the top leading causes of cancer death in both males and females worldwide [1]. Small cell lung cancer (SCLC) accounts for around 13% of all lung cancers [2]. However, SCLC is the more aggressive and deadlier form of lung cancer due to a predisposition for rapid growth, early metastasis, and acquired therapeutic resistance [3–5]. Thus, SCLC can be found rarely localized at diagnosis [6]. Therefore, discovering potential biomarkers for SCLC will be crucial in advancing our overall knowledge of the molecular basis underlying SCLC tumorigenesis in hopes to provide a promising new chemical modality with clinical relevance.

Emerging studies have demonstrated that mutations and dysregulations in the activity of epigenetic factors are collectively associated with the pathogenesis of a diverse abundance of human cancers [7], including SCLC [8–10]. In our recent studies, we have identified and characterized a unique enhancer-bound epigenetic machinery comprised of the deubiquitinase BAP1 complex and bromodomain-containing protein BRD4, linked by a tissue-specific BAP1 complex subunit known as additional sex combs-like protein ASXL3. The BAP1/ASXL3/BRD4 epigenetic axis determines enhancer activity and gene expression critical for cell viability in SCLC cells [11].

Initially, BAP1 was identified as a major histone H2AK119 deubiquitinase in Drosophila [12]. In mammalian cells, BAP1 functions as a multi-protein complex that contains as many as ten different subunits [11, 13–18], including ASXL1/2/3, FOXK1/2, MBD5/6, HCFC1, and OGT. In mammalian cells, the BAP1 complex has been characterized as a general transcriptional activator and facilitates transcription of genes by antagonizing Polycomb Repressive Complex 1 (PRC1) function, which deposits histone H2AK119 mono-ubiquitination [19, 20]. Besides targeting just histone substrates, BAP1 can also function as a deubiquitinase of non-histone proteins, such as HCFC1 [14], KLF5 [21], INO80 [22], DNMT1 [23], IP3R3 [24], and PGC1-α [25], most of which have been reported as key regulators in human cancers.

Dysregulations and mutations within the *BAP1* gene have been identified as drivers in many human cancers, such as uveal melanoma [26–28], mesothelioma [29], clear-cell renal cell carcinoma [30, 31], leukemia [32], and breast cancer [21]. Initially, loss of BAP1 has been linked to myeloid transformation in animal models [16]. However, emerging studies have uncovered the dual roles of BAP1 or the BAP1 complex and how this can impact tumorigenesis in different human cancers. For instance, BAP1 has been demonstrated to stabilize an oncogenic transcription factor KLK5 in breast cancer [21], promoting breast cancer progression and metastasis. Furthermore, in mutant ASXL1-driven leukemia models, reducing BAP1’s catalytic activity has been shown to inhibit leukemia development and extend the survivability of animals [33]. Therefore, in our recent studies, we have identified a first-in-class BAP1 inhibitor by utilizing a high throughput small-molecule screening and tested its activity, efficacy, and specificity both in vitro and in vivo [34]. In this current study, we identified and characterized a next-generation BAP1 inhibitor (iBAP-II) with higher activity and specificity, and further determined its impact on BAP1-dependent transcriptional programming and SCLC cell viability in vitro and in vivo.

## Results

### Identification and characterization of iBAP-II as a next-generation BAP1 inhibitor

**Fig. 1:**
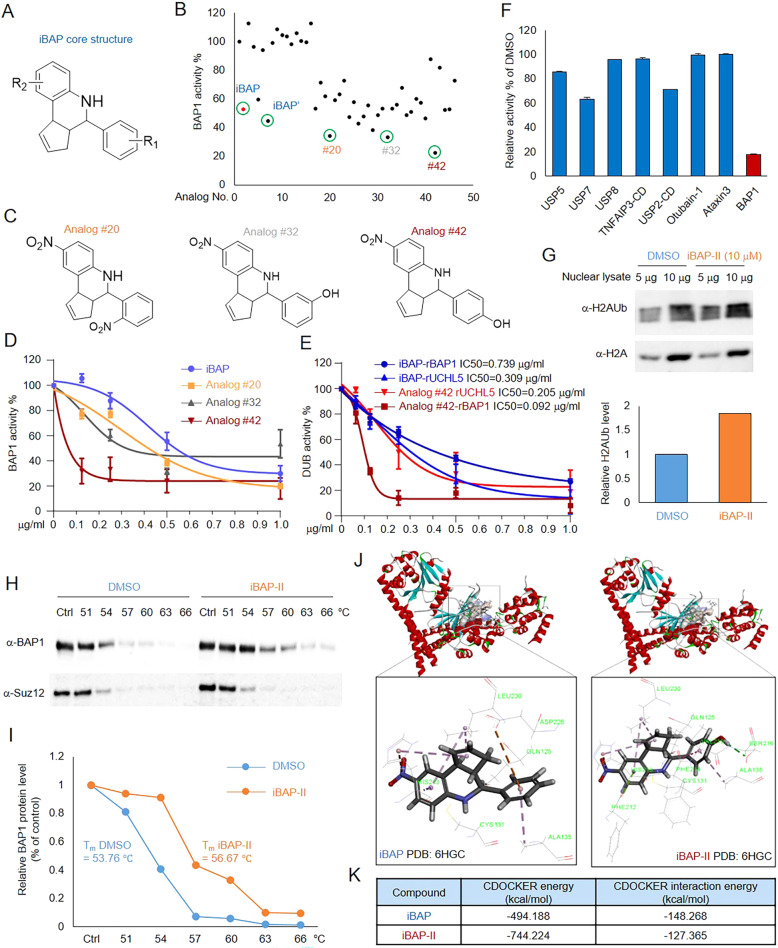
Identification and characterization of iBAP-II as a next-generation BAP1 inhibitor. **A** The core structure of iBAP compound. **B** 44 new analogs of iBAP were designed and synthesized based on the core structure of iBAP. The inhibitory effect of all the compounds were determined by Ub-AMC assay. Commercially available iBAP (iBAP: purity >90%) and in-house synthesized iBAP (iBAP’: purity >95%) were used as positive controls, DMSO was used as the negative control, *n* = 2. **C** Structures of Analogs #20, #32, and #42. D) Dose-dependent inhibition by iBAP and three analogs from **C** were determined by Ub-AMC assay, *n* = 3. **E** Dose-dependent inhibition of BAP1 and UCHL5 activity by iBAP and Analog #42 by Ub-AMC assay, *n* = 3. The IC50 for each condition was provided. **F** The inhibitory effects of different DUBs’ activity by Analog #42 (1 μg/ml) using Ub-AMC assay, *n* = 3. **G** NCI-H1963 cells were treated with iBAP-II (10 μM) for 24 h, the protein levels of H2AK119Ub were determined by western blot (upper panel), and quantified by ImageJ (lower panel). Nuclear extracts of SCLC cells were incubated at different temperatures for 5 min in the presence of either DMSO or iBAP-II (1 μg/ml). The protein levels of BAP1 in the soluble fraction were determined by western blot, *n* = 2 (**H**) and quantified by ImageJ (**I**). **J** Illustrative molecular docking of iBAP and iBAP-II into the three-dimensional X-ray structure of Calypso (the BAP1 ortholog from Drosophila melanogaster, PDB: 6HGC) was carried out using the Discovery Studio (version 4.5) as implemented through the graphical user interface CDOCKER protocol. **K** The CDOCKER energy and CDOCKER interaction energies for the top ranked binding poses of iBAP/iBAP-II obtained after conducting the molecular docking studies on Calypso (PDB: 6HGC) using the CDOCKER algorithm in Discovery Studio 4.5.

BAP1-dependent cell growth and viability have been reported in multiple cancer models in vitro and in vivo. In addition, the deubiquitinase activity of BAP1 has been linked to BAX- or BAK- dependent apoptosis in RING1B highly expressed cells. Therefore, in our previous studies, by utilizing high throughput small-molecule screening, we have identified the first-in-class inhibitor against BAP1’s catalytic activity, named iBAP (Supplementary Fig. 1A), with the IC50 in the range of 0.5–1 μM in vitro [34]. To further increase the activity, efficacy, and specificity of the first-generation inhibitor, we designed and synthesized 44 additional analogs of iBAP based on the core structure of this compound (Fig. 1A, Supplementary Fig. 1B, Supplementary Table 1), and further determined the inhibition activity of these indicated analogs by performing an Ub-AMC assay with the original compound iBAP and DMSO as the positive and negative controls, respectively (Fig. 1B).

As a result, we have identified several new analogs, which have either –NO2 or –OH modifications at the –R1 position, that are more efficient than the original iBAP small-molecule inhibitor (Fig. 1B, C). Specifically, we have identified the Analog #42 is the best compound that could most efficiently inhibit BAP1 activity in vitro (Fig. 1D). In addition, in comparison to iBAP, Analog #42 has a higher affinity for BAP1 than for the closest UCH family member, UCHL5 (Fig. 1E), and other deubiquitinases, such as USP5, USP7, USP8, TNFAIP3-catalytic domain, USP2-catalytic domain, Otubain-1, and Ataxin3 (Fig. 1F). Therefore, we named Analog #42 as iBAP-II in the subsequent studies. To demonstrate a functional inhibition of BAP1 by iBAP-II in vivo, we treated human SCLC cell line NCI-H1963 cells with iBAP-II, followed by western blot to determine the protein levels of histone H2AK119Ub. As shown in Fig. 1G, we found an increase of H2AK119Ub upon iBAP-II treatment. To further validate the target engagement effects of iBAP-II in cells, we employed a cellular thermal shift assay by determining the solubility of endogenous BAP1 protein in the presence of either DMSO or iBAP-II upon gradient heat treatment. As shown in Fig. 1H, we found iBAP-II could increase the solubility of BAP1 upon heat treatment (ΔT_m_ = 2.91 °C) (Fig. 1I).

To understand the binding mode of the iBAP/iBAP-II with BAP1 protein, we carried out the molecular docking of candidate compounds with the published crystal structure of Calypso (PDB: 6HGC), the Drosophila homolog of human BAP1, which is highly conserved when compared to human BAP1 (55% identical and 71% similar amino acids in the structured regions) [35]. For instance, Calypso’s catalytic triad, which includes CYS131, HIS213, and ASP228, is the same as that seen in human BAP1 (Supplementary Fig. 1C). Based on our theoretical docking model (Fig. 1J, K), both iBAP and iBAP-II could be embedded into the catalytic domain of Calypso via hydrogen bonds, π-cation interactions, π-σ interactions and π-alkyl interactions with amino acid residues of Calypso. Moreover, our model suggested the phenolic hydroxyl group of iBAP-II could form an additional hydrogen bond with SER216 (Fig. 1J; Supplementary Fig. 1D, E), which may contribute to the enhanced potency of iBAP-II in our biochemistry studies. Interestingly, based on our additional theoretical docking model, iBAP-II failed to dock into the catalytic domain of UCH-L5, the closest UCH family member with BAP1, due to the differences in the structural binding region of the active site [35] (Supplementary Fig. 1F), which is in agreement with our biochemical studies that illustrate a potent and selective interaction between iBAP-II and Calypso. Finally, we cross-linked iBAP-II to the epoxy-activated-agarose, and further incubated it with either recombinant BAP1 protein purified from bacteria or whole nuclear extract from NCI-H1963 cells. As shown in Supplementary Fig. 1G, we found the agarose beads cross-linking with iBAP-II could successfully enrich BAP1 protein in both conditions. These results further demonstrated the direct interaction between iBAP-II and BAP1 protein.

### Inhibition of BAP1 reduces ASXL3 protein stability in small cell lung cancer cells

**Fig. 2:**
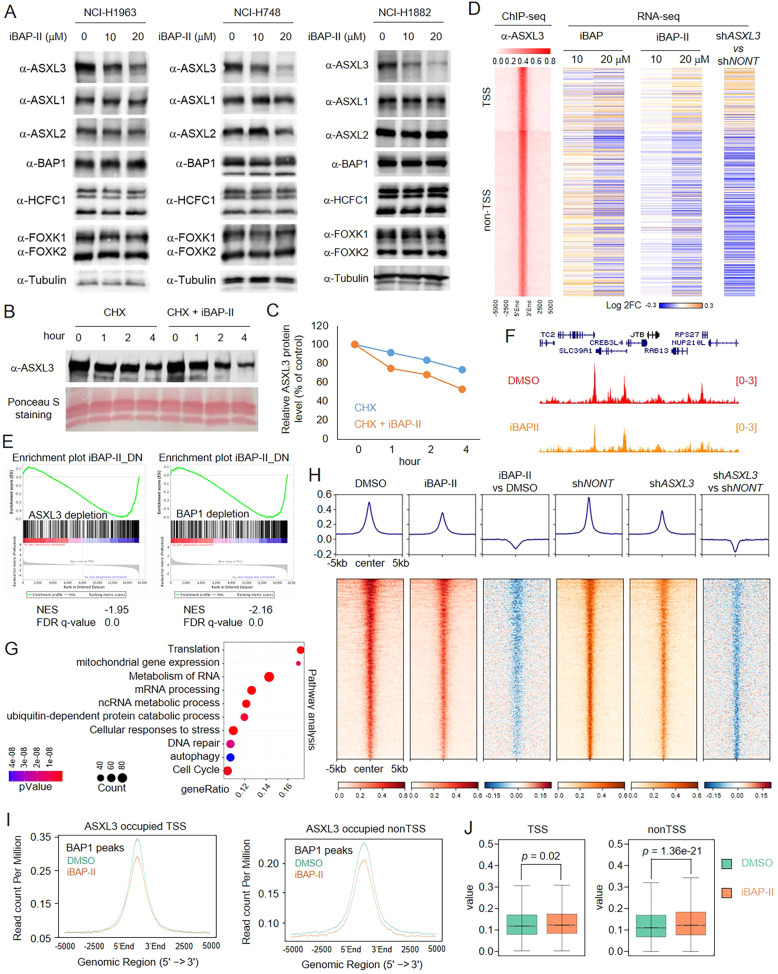
Inhibition of BAP1 reduces ASXL3 protein stability in small cell lung cancer cells. **A** Three different human SCLC cell lines (NCI-H1963, NCI-H748, and NCI-H1882) were treated with iBAP-II (0, 10, and 20 μM) for 24 h. The protein levels of ASXL1, ASXL2, ASXL3, BAP1, HCFC1, FOXK1, and FOXK2 were determined by western blot. β-tubulin was used as the internal control. Human SCLC cell line NCI-H1963 cells were treated with 50 μg/ml cycloheximide (CHX) at different times in the presence of either DMSO or iBAP-II. The ASXL3 protein levels were determined by western blot (**B**) and further quantified by ImageJ (**C**), *n* = 2. **D** The log2 fold change of the expression levels of genes nearby ASXL3 peaks grouped as TSS and nonTSS regions annotated by Homer in ASXL3-depleted cells, and cells treated with either iBAP or iBAP-II, *n* = 2. **E** Gene Set Enrichment Analysis (GSEA) of iBAP-II gene signature enrichment in ASXL3 or BAP1-depleted conditions. A total of 383 downregulated genes in iBAP-II (20 μM) versus DMSO treatment (adj.*p* < 0.01, logFC < −1) is defined as the iBAP signature gene set. GSEA of iBAP signature genes in ASXL3-depleted cells compared with control cells by shRNA. GSEA of iBAP signature genes in BAP1-depleted cells compared with control cells by sgRNA. **F** Representative track example that shows BAP1 occupancy in NCI-H1963 cells treated with either DMSO or iBAP-II. **G** Pathway analysis shows the most significant signaling pathways enriched in genes nearest to the loss of BAP1 peaks (*n* = 1999) induced by iBAP-II treatment. **H** ChIP-Seq analysis representing 70% (2470/3529) of all BAP1 peaks from BAP1 chromatin occupancy being collectively reduced upon iBAP treatment. Occupancy and log2FC heatmaps of BAP1 in DMSO and iBAP-II conditions are centered on these BAP1 peaks (*n* = 2470). When comparing the same ranking of peaks, BAP1 occupancy also decreased from ASXL3 depletion via shRNA. Peaks were sorted based on the mean signals from DMSO and iBAP-II samples in each row. **I** The average plot shows BAP1 levels at ASXL3-occupied TSS (left) and nonTSS (right) regions. **J** The box plot shows the H2AK119Ub levels between DMSO and iBAP-II treated NCI-H1963 cells.

The ASXL genes (ASXL1, ASXL2, and ASXL3) encode for mutually exclusive core subunits within the BAP1 complex, which specifically interact with the C-terminus of BAP1. Interestingly, the ASXL subunit could also be stabilized by BAP1 in a catalytic-dependent manner [32]. Therefore, to determine the impact of BAP1 inhibition on the protein stability of ASXL1-3, we treated three different SCLC cell lines, NCI-H1963, NCI-H748, and NCI-H1882 cells with varying concentrations of iBAP-II for 24 h. Treatment with iBAP-II significantly reduced ASXL3 protein levels, while there being no significant changes to ASXL1 and ASXL2 protein levels (Fig. 2A, Supplementary Fig. 2A). To determine whether iBAP-II treatment reduced the protein stability of ASXL3, we treated NCI-H1963 cells with cycloheximide (CHX) in the presence of either DMSO or iBAP-II using different exposure times. As shown in Fig. 2B, C, we found treatment with iBAP-II lead to a faster degradation of ASXL3 protein. Consistent with this result, we discovered that ASXL3 protein is less stable than ASXL1 and ASXL2 in SCLC cell lines (Supplementary Fig. 2B, C).

In our previous studies, we have demonstrated that genetic depletion of ASXL3 leads to a reduction of BAP1 occupancy at the chromatin. Therefore, to determine how iBAP-II regulates transcription in SCLC cells via ASXL3 protein, we conducted RNA-seq in NCI-H1963 cells treated with different concentrations of either iBAP or iBAP-II (Supplementary Fig. 2D) and further compared the gene expression profile between iBAP/iBAP-II treated cells and ASXL3-depleted cells. As shown in Fig. 2D, the log2 fold-change heatmap of metagene analysis showed that the gene expression patterns between iBAP-II treated cells and ASXL3-depleted cells resembled each other. To extend this observation on a global scale, we employed Gene Set Enrichment Analysis (GSEA) of iBAP-II gene signature enrichment in ASXL3 or BAP1-depleted conditions (Fig. 2E). The iBAP-II signature genes were significantly enriched in downregulated genes with ASXL3 or BAP1 depletion.

Our previous studies have demonstrated that ASXL3 is responsible for the chromatin recruitment of BAP1 in SCLC cells [14]. To determine whether loss of ASXL3 expression induced by iBAP-II treatment affects BAP1 occupancy, we conducted BAP1 ChIP-seq in NCI-H1963 cells treated with either DMSO or iBAP-II. In total 2691 BAP1 peaks in cells treated with DMSO and 1530 peaks in iBAP-II treated cells were called (Fig. 2F). Pathway analysis has shown that the BAP1 peaks that are localized close to the genes involved in mRNA processing, metabolism, and cell cycle are lost upon iBAP-II treatment (Fig. 2G), which is consistent with the comprehensive RNA-seq pathway analysis shown in Supplementary Fig. 2E. To determine whether the reduced BAP1 peaks are ASXL3-dependent, we compared the BAP1 peaks between iBAP-II treated cells and ASXL3-depleted cells. As shown in Fig. 2H, I, we found that the pattern of BAP1 peaks in iBAP-II treated cells versus ASXL3-depleted cells is very similar, and the reduction of BAP1 peaks at ASXL3-occupied nonTSS regions induced a significant increase of histone H2AK119Ub levels (Fig. 2J).

### BAP1 inactivation suppresses ASCL1 expression

**Fig. 3:**
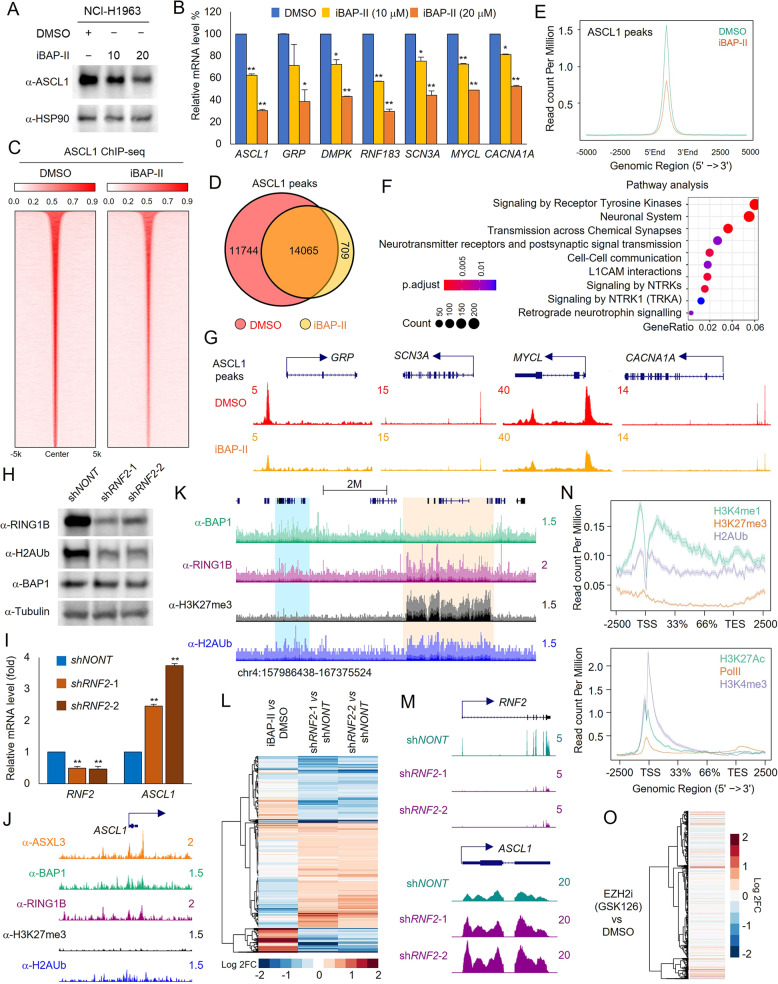
BAP1 inactivation suppresses ASCL1 expression. **A** NCI-H1963 cells were treated with iBAP-II for 24 h. The protein levels of ASCL1 were determined by western blot. HSP90 was used as the internal control, *n* = 2. **B** The mRNA levels of ASCL1, GRP, DMPK, RNF183, SCN3A, MYCL, and CACNA1A were determined by real-time PCR in NCI-H1963 cells treated with either 10 or 20 μM of iBAP-II. DMSO was used as the negative control, *n* = 3. **C** The occupancy heatmaps show ASCL1 binding levels to chromatin after iBAP-II treatment. The Venn-diagram (**D**) and average plot (**E**) show the ASCL1 levels at chromatin in NCI-H1963 cells treated with either DMSO or iBAP-II. **F** Pathway analysis shows the most significant signaling pathways enriched in genes nearest to the loss of ASCL1 peaks (*n* = 11,744) induced by iBAP-II treatment. **G** Representative track example that shows ASCL1 occupancy between NCI-H1963 cells treated with either DMSO or iBAP-II. **H** NCI-H1963 cells were transduced with two distinct RING1B shRNAs. The protein levels of RING1B, H2AK119Ub, and BAP1 were determined by western blot. β-tubulin was used as the internal control, *n* = 2. **I** The mRNA levels of ASCL1 were determined by real-time PCR in NCI-H1963 cells transduced with either non-targeting gRNA or two distinct RING1B shRNAs, *n* = 3. **J** The representative tracks show the chromatin occupancy of ASXL3, BAP1, RING1B, H3K27me3, and H2AK119Ub levels at the *ASCL1* gene locus in NCI-H1963 cells. **K** Representative tracks show the occupancy of BAP1, RING1B, H3K27me3, and H2AUb levels at chromatin. **L** RNA-seq was performed with NCI-H1963 cells treated with iBAP-II or transduced with non-targeting shRNA and two different RING1B gRNAs, *n* = 2. **M** The representative tracks show the expression levels of *RNF2* and *ASCL1* in cells transduced with either non-targeting gRNA or two distinct RING1B shRNAs. **N** The average plots show the levels of H3K4me1 (GSE145028), H3K27me3 (GSE164247), H2AK119Ub (upper), H3K27Ac (GSE145028), H3K4me3 (GSE164247), and Pol II (lower) at the gene body of all 739 genes that were downregulated in iBAP-II treated cells and upregulated in RING1B-depleted cells. **O** RNA-seq was performed with cells treated with either DMSO or EZH2 inhibitor GSK126 (2 μM) for 96 h, *n* = 2. The Log2FC heatmap shows the gene expression changes in the same order as **L**.

ASCL1 is one of the master transcription factors of small cell lung carcinoma and defines SCLC-A subtype—the most abundant representative of the subtype population in human SCLC. Consequently, depletion of ASCL1 abolishes SCLC tumor growth both in vitro and in vivo [36, 37]. Based on our RNA-seq data analysis in Supplementary Fig. 2D, E, we found ASCL1 was one of the top downregulated genes upon iBAP-II treatment. To determine whether treatment of iBAP-II could also reduce ASCL1 at the protein level, we determined the protein levels for ASCL1 by western blot analysis in cells treated with different concentrations of iBAP-II. Indeed, treatment with iBAP-II reduced ASCL1 protein expression in NCI-H1963 cells (Fig. 3A), along with the mRNA levels involving a handful of known ASCL1 transcriptional targeted genes, such as *GRP*, *DMPK*, *RNF183*, *SCN3A*, *MYCL*, and *CACNA1A* (Fig. 3B). Moreover, the downregulation of ASCL1 mRNA and protein levels by iBAP-II treatment was also validated in other SCLC cell lines NCI-H748 and NCI-H1882 (Supplementary Fig. 3A, B). Consistently, as a component of the BAP1/ASXL3/BRD4 epigenetic axis described previously, inhibition of BRD4 by utilizing three different BET inhibitors has also shown similar results in reducing *ASCL1* expression (Supplementary Fig. 3C).

In addition, we depleted BAP1 with CRISPR sgRNA to validate the above results. Depletion of BAP1 with CRISPR sgRNAs also reduced *ASCL1* expression levels in the same cell line (Supplementary Fig. 3D, E). Then, we asked whether inhibition of BAP1 would reduce ASCL1 function at the chromatin level by conducting ASCL1 ChIP-seq in cells treated with DMSO or iBAP-II. As a result, we detected 25,809 peaks in cells treated with DMSO and only 14,774 BAP1 peaks in iBAP-II treated cells (Fig. 3C–E). Indeed, genes localized close to ASCL1 peaks that were lost upon iBAP-II treatment (11,744 peaks as shown in Fig. 3D) were significantly enriched in multiple neural functional pathways (Fig. 3F). Furthermore, track examples showed a dramatic reduction of ASCL1 occupancy at the promoter regions of its targeted genes, such as *GRP*, *SCN3A*, *MYCL*, *CACNA1A*, *RNF183*, and *DMPK* (Fig. 3G and Supplementary Fig. 3F).

As far as we know, BAP1 is a major deubiquitinase targeting H2AK119Ub, which is solely deposited by the PRC1 complex through its two paralogous enzymes, RING1A and RING1B. RING1B deletion could significantly improve cell viability of BAP1 −/− cells, while deletion of RING1A provided little to no benefit [20]. Therefore, we knocked down RING1B with two distinct shRNAs (Fig. 3H) and asked whether loss of RING1B could activate *ASCL1* gene expression. As shown in Fig. 3I, we have detected a significant increase in *ASCL1* expression in cells transduced with two different RING1B shRNAs. Consistent with this result, we have detected BAP1 and RING1B co-occupancy at *ASCL1* gene locus (Fig. 3J). However, there was a significant enrichment of histone H2AK119Ub, but not H3K27me3, detected at the *ASCL1* gene locus (Fig. 3J), as well as other loci across the genome (Fig. 3K). This result leads us to further compare the global gene expression profiles between iBAP-II treated cells and RING1B-depleted cells to determine the transcriptional overlap. As a result, we found a significant number of genes (*n* = 739) that are upregulated by RING1B depletion, and these genes could be repressed by iBAP-II treatment (Fig. 3L, M). Most of those genes are enriched in the cell cycle and DNA replication pathways (Supplementary Fig. 3G). Consistent with the result we showed at *ASCL1* gene locus (Fig. 3J), we detected a co-occupancy of BAP1 and RING1B at the open chromatin loci of the 739 genes (Supplementary Fig. 3H, I). Interestingly, a significant enrichment of H3K4me1, H3K27Ac, H3K4me3, Pol II, and H2AK119Ub levels were detected, whereas H3K27me3 levels were devoid at the 739 gene loci (Fig. 3N). These results suggested that commonly targeted genes by BAP1 and RING1B may be independent of PRC2 function (via H3K27me3 activity of EZH2). Indeed, treatment with EZH2 inhibitor could not rescue the expression of the indicated 739 genes (Fig. 3O) or *ASCL1* expression in SCLC cells (Supplementary Fig. 3J).

### Efficacy of iBAP-II in SCLC tumor growth and viability

**Fig. 4:**
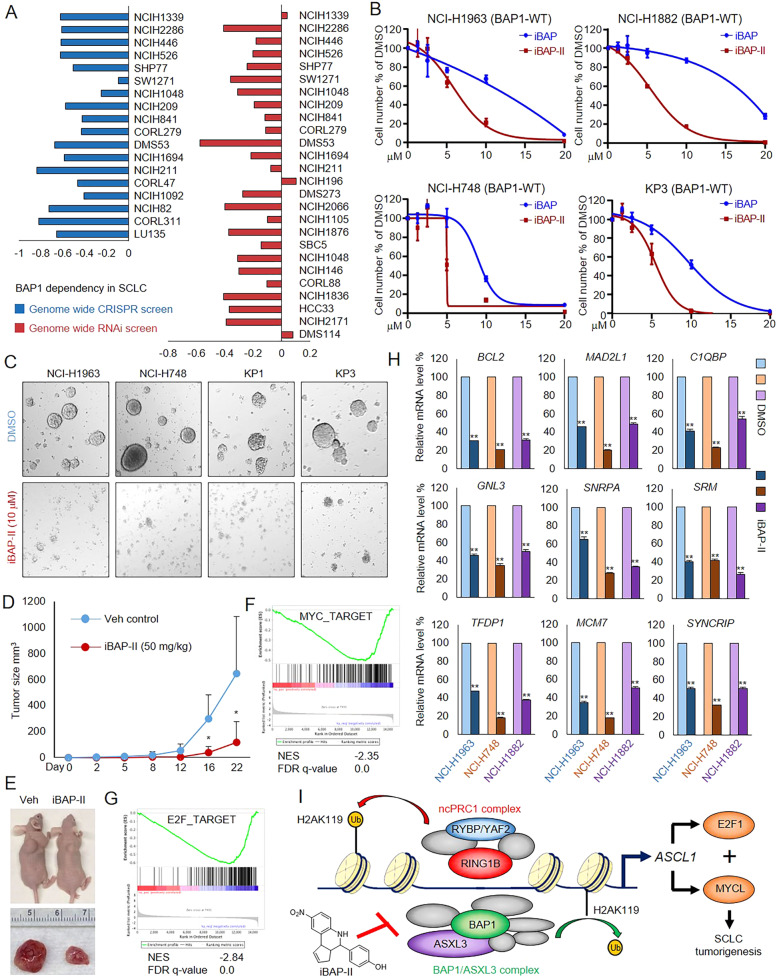
Efficacy of iBAP-II in SCLC tumor growth and viability. **A** The BAP1-dependency results in all SCLC cell lines were retrieved from the Cancer Dependency Map (DepMap). The left panel shows BAP1-dependency determined by genome-wide CRISPR screening. The right panel shows BAP1-dependency determined by genome-wide RNAi screening. **B** Four different SCLC cell lines NCI-H1963, NCI-H1882, NCI-H748, and KP3 (mouse) cells were treated with various concentrations of iBAP-II for 72 h. The cell number was determined by cell counting assay, *n* = 3. **C** Representative photographs show the colony formations in four different types of SCLC cells treated with either DMSO or iBAP-II for 72 h. **D** 5.0 × 10^5^ of mouse SCLC KP3 cells were inoculated into the right flank of nude mice. Two weeks after inoculation, vehicle (*n* = 5) or 50 mg/kg of iBAP-II (*n* = 5) was administered daily by intraperitoneal (IP) injections, and the tumor growth was measured every 4 days using a calibrated caliper. A two-tailed unpaired Student’s *t*-test was used for statistical analysis. **P < 0.01; *P < 0.05. **E** Images for representative tumor tissue from each mouse were taken at the end of the experiment. **F**, **G** GSEA analyses show the most enriched gene expression signatures correlated with downregulated genes in iBAP-II treated cells. **H** The mRNA levels of the MYC pathway genes *BCL2*, *MAD2L1*, *C1QBP*, *GNL3*, *SNRPA*, *SRM*, *TFDP1*, *MCM7*, and *SYNCRIP* were determined by real-time PCR in three different SCLC cells treated with either DMSO or iBAP-II for 24 h. **I** The graphic model shows the epigenetic balance between the BAP1 and ncPRC1 complexes in determining *ASCL1* gene expression and ASCL1-dependent transcriptional signatures in SCLC.

To determine the dependency of BAP1 in a broad panel of SCLC cell lines, we retrieved the genome-wide CRISPR screening and RNAi screening results in different SCLC cell lines from the Cancer Dependency Map (DepMap). As shown in Fig. 4A, a vast majority of the tested SCLC cell lines are sensitive to BAP1 depletion in vitro. To confirm the BAP1-dependent tumor growth in vivo, we depleted BAP1 in mouse SCLC KP1 cells by CRISPR and determined the cell growth both in vitro and in vivo. As shown in Supplementary Fig. 4A–D, we found loss of BAP1 significantly reduced tumor progression and cell viability both in vitro and in vivo using two distinct BAP1 gRNAs.

Therefore, to determine the impact of iBAP-II on SCLC cell viability, we treated four different BAP1-WT SCLC cell lines (NCI-H1963, NCI-H748, NCI-H1882, and KP3 cells) with either DMSO, iBAP, or iBAP-II. An additional BAP1-null SCLC cell line, NCI-H226, was also included as a negative control (Supplementary Fig. 4E). As a result, we found both of the inhibitors efficiently reduced cell viability in vitro. Notably, iBAP-II exhibited a more potent inhibition activity on all four different cell lines than iBAP (Fig. 4B). Interestingly, the BAP1-null cell line NCI-H226 showed less sensitivity towards either iBAP or iBAP-II treatment (Supplementary Fig. 4F, G). In addition, we observed consistent results from in vitro colony formation ability in both human and mouse SCLC cell lines when treated with iBAP-II versus DMSO (Fig. 4C). To test the effects of BAP1 inhibition on SCLC progression in vivo, we established a mouse xenograft model by inoculating mouse KP3 SCLC (p53/Rb mutant) cells into the right flank of athymic nude mice. Two weeks after the transplantation, we treated the animals with either vehicle or iBAP-II (50 mg/kg/d). We found BAP1 inhibition by iBAP-II significantly delayed the disease progression in our SCLC xenograft model (Fig. 4D, E).

To determine the major signaling pathways impacted by iBAP-II in SCLC cells, we introduced GSEA pathway analysis and compared the iBAP-II targeted genes obtained from NCI-H1963 cells and previously defined set of genes. As shown in Fig. 4F, G, we found MYC_targets and E2F_targets are the top 2 pathways that were significantly enriched in iBAP-II downregulated genes. To confirm this, we further validated a number of MYC and E2F targeted genes in three different human SCLC cell lines (NCI-H1963, NCI-H1882, and NCI-H748) treated with either DMSO or iBAP-II using real-time PCR. As a result, we found consistent repression of the indicated genes (e.g., *BCL2*, *MAD2L1*, *C1QBP*, *GNL3*, *SNRPA*, *SRM*, *TFDP1*, *MCM7*, *SYNCRIP*, *SNRPG*, *MRPL23*, *HNRNPR*, *LSM7*, and *ODC1*) by iBAP-II in all three SCLC cell lines (Fig. 4H, Supplementary Fig. 4H).

Finally, to evaluate the potential clinical relevance of BAP1 targeted genes in patient samples, we retrieved the RNA-seq data from both normal lung tissues (*n* = 7) and SCLC patient tissues (*n* = 79) and further isolated the genes that are significantly dysregulated in tumor samples [38]. GSEA plot shows SCLC downregulated signature genes are significantly enriched in upregulated genes upon iBAP-II treatment, while SCLC upregulated signature genes are significantly enriched in downregulated genes with iBAP-II treatment (Supplementary Fig. 4I). This result indicated that the oncogenic transcriptional signatures in tumors could be potentially suppressed by BAP1 inhibition. By regulating BAP1-dependent transcriptional signatures, such as ASCL1/MYCL/E2F signaling, our studies highlight the potential to target BAP1’s activity using our next-generation iBAP inhibitor as a novel form of SCLC therapy (Fig. 4I). Moreover, we confirmed the establishment of an epigenetic dynamic interplay between the BAP1/ASXL3 complex and the opposing ncPRC1 complex, which could potentially be targeted for developing other therapeutics in order to increase the effectiveness against SCLC tumor progression.

## Discussion

Initially, BAP1 has been characterized as a tumor suppressor in animal models [16]. It has also been shown that loss of BAP1 may lead to myeloid transformation in vivo, suggesting that BAP1 may function as a tumor suppressor in the hematopoietic system. In addition, mutations within BAP1’s catalytic domain have been detected in different types of human cancers such as uveal melanoma and have been demonstrated as loss-of-function mutations [27]. However, emerging results from various studies have shown that hyper-activated BAP1 may also function as an oncogene in vitro and in vivo. For instance, hyper-activation of BAP1 induced by ASXL1 gain-of-function mutations has been demonstrated to be drivers in leukemia of an animal model [32, 33, 39, 40]. Therefore, reducing BAP1 levels and activity could prevent ASXL1 truncation-driven myeloid malignancies [33]. In human breast cancer, the oncogenic transcription factor KLF5 (highly expressed in basal-like breast cancer) could be stabilized by BAP1, which further promotes breast cancer cell development both in vitro and in vivo [21]. As a result, depletion of BAP1 inhibits breast cancer tumorigenicity and lung metastasis. Overall, these studies implied that BAP1 might have dual functionality and context-dependent roles in tumorigenesis.

To determine the catalytic activity of BAP1 involved in cell viability, Dixit’s lab generated the first conditional knock-in mice that express catalytically inactive BAP1 (C91A) instead of wild-type BAP1 after tamoxifen treatment. Interestingly, conditional knock-in of a catalytically-dead version of BAP1 strongly repressed the expression of pro-survival genes (e.g., *BCL2* and *MCL1*), leading to BAX- or BAK-dependent apoptosis [20]. These results further supported the feasibility of developing a small-molecule inhibitor against BAP1’s activity as a new SCLC therapy. Therefore, in our most recent studies we have identified the first-in-class inhibitor against BAP1’s catalytic activity by utilizing a high throughput small-molecule screening [34]. We then characterized this iBAP inhibitor as having an anti-leukemia effect both in vitro and in vivo.

Our current studies have demonstrated that BAP1 functions as a common essential factor in most SCLC cell lines. Depletion of BAP1 significantly reduced SCLC cell growth in the cell-based and animal xenograft models. Thus, by applying additional chemical modifications and small-scale screenings, we have identified a next-generation BAP1 inhibitor, which shows a more robust activity, specificity, and anti-tumor effects than the original version. Moreover, our previous studies have characterized a unique epigenetic axis comprised of BAP1 and the bromodomain protein BRD4, which are bridged together by ASXL3 subunit within the BAP1 complex [11]. We further demonstrated that ASXL3 is highly expressed and also essential for cell viability in SCLC cells by controlling the transcription of a number of SCLC essential genes such as *PAX9*, *WDR72*, *CDH7*, *BCL2*, etc., which are also direct transcriptional target of BAP1 [11, 41]. Interestingly, we found inhibition of BAP1 dramatically destabilizes ASXL3 in SCLC cells and further disrupts the BAP1/ASXL3/BRD4 epigenetic axis. Although iBAP-II shows a stronger effect and higher selectivity than the original version, we did however notice that iBAP-II could also inhibit cell growth in BAP1-null SCLC cells at high concentrations. This finding suggests that iBAP-II may also hit other oncogenic targets that are essential for SCLC cell viability. Future studies will be focused on improving the specificity of iBAP serial compounds.

It has been known that the activity of BAP1 is critical for the maintenance of the protein stability of the additional sex combs-like protein ASXL1-3, including CTD-truncated ASXL1 variant in cancer [19, 32]. However, in multiple SCLC cells lines we have tested, the ASXL3 protein is less stable than ASXL1 and ASXL2 proteins upon iBAP-II treatment. Since the ASXL3 protein is a tissue-specific additional sex combs-like protein, and is twice larger than ASXL1/2, it is possible that ASXL3 also has its unique transcriptional/post-transcriptional regulatory control in SCLC cells. Interestingly, it may very well be worth investigating the identification of any additional degron domains within ASXL3 that are not present ASXL1/ASXL2 in future studies.

It has been demonstrated by several groups that the balance between the BAP1 and PRC1 complexes can determine the stages and dynamics of histone H2AK119Ub levels, and the precise regulation is conserved in metazoans from Drosophila to mammals [19, 20]. As far as we know, the mammalian PRC1 complexes can be divided into two main categories—canonical and non-canonical complex variants, based on their dependency on PRC2-deposited H3K27me3 histone marks [42, 43]. Interestingly, in SCLC cells, we found a very low enrichment of H3K27me3 marks on the genes that are BAP1 and PRC1 co-dependent, suggesting that at least in SCLC cells, BAP1 may function opposite to ncPRC1 [44, 45].

ASCL1 is a neuroendocrine lineage-specific oncogenic driver of SCLC and is highly expressed in SCLC and lung tumors of neuroendocrine origin [46]. Therefore, based on the unique tissue specificity and expression patterns, ASCL1 has been used to define the SCLC-A subtype, which accounts for ~70% of all human SCLC. In addition, it has been known that *ASCL1* is a bivalent gene decorated with PRC1/2 at its CpG-island promoter in mouse embryonic stem cells [47]. However, in our current study, we did not see obvious H3K27me3 signals at the *ASCL1* gene loci in SCLC cells, and treatment of EZH2 inhibitor does not induce the expression of *ASCL1* gene. These results suggest critical epigenetic states regulated by BAP1 and ncPRC1 in human SCLC and provide potential therapeutic targets for future drug development and clinical SCLC therapy.

## Materials and methods

### Antibodies and reagents

ASCL1 (#43666), BAP1 (#13271S), H3K27ac (#8173S), H3K4me1 (#5326S), H3K4me3 (#9751), H3K27me3 (#9733), H2AK119Ub (#8240), ASXL2 (#71257), and RING1B (#5694) were purchased from Cell Signaling Technology (CST) company. HSP90 (sc-7947) was purchased from Santa Cruz. Tubulin antibody (E7) was purchased from Developmental Studies Hybridoma Bank. FOXK1 (A301-727A), FOXK2 (A301-729A), and HCFC1 (A301-399A) antibodies were purchased from Bethyl-Laboratories. ASXL1 and ASXL3 antibodies were generated as previously described [11]. EZH2 inhibitor GSK126 was purchased from Cayman (15415).

### Cell lines

HEK293T cells were obtained from ATCC, and then maintained with DMEM (Gibco, Gaithersburg, MD) containing 10% FBS (Sigma). The human SCLC cell lines were obtained from ATCC. NCI-H748, NCI-H1963, and NCI-H226 were maintained with ATCC-formulated RPMI-1640 medium containing 10% FBS (Sigma). NCI-H1882 cells were maintained with ATCC-formulated DMEM/F12 cell culture media containing 10% FBS (Sigma). Mouse KP1 and KP3 were maintained with ATCC-formulated RPMI-1640 medium containing 10% FBS (Sigma).

### General chemistry method

All reagents were purchased from commercial sources and used as received without further purification. Reactions were monitored by thin-layer chromatography (TLC) on Merck silica gel 60 F254 plates visualized under ultraviolet light (254 nm). Compounds were purified using flash column chromatography over silica gel (200–300 mesh). 1H NMR spectra were recorded on a Bruker AV-300 spectrometer at room temperature with tetramethyl silane as an internal standard. Chemical shifts were reported in ppm (δ). High-resolution mass spectrometry was obtained on a Q-tof high-resolution mass spectrometer. HPLC analysis showed that all tested compounds have >95% purity.

### Molecular docking method

Molecular docking of iBAP and iBAP-II into the three-dimensional X-ray structure of Calypso (the BAP1 ortholog from Drosophila melanogaster, PDB: 6HGC) was carried out using the Discovery Stutio (version 4.5) as implemented through the graphical user interface CDOCKER protocol. Calypso is highly conserved compared with human BAP1. Moreover, Calypso catalytic triad which includes CYS131, HIS213, and ASP228 is the same as that in human BAP1. The geometry of the Calypso catalytic triad is consistent with its substrate-free state. Therefore, the catalytic triad was selected to define binding site. The three-dimensional structures of the compounds iBAP and iBAP-II were constructed using ChemBio 3D Ultra 14.0 software [Chemical Structure Drawing Standard; Cambridge Soft corporation, USA (2014)], then they were energetically minimized by using MMFF94. The crystal structures of Calypso were retrieved from the RCSB Protein Data Bank (http://www.rcsb.org). All bound waters and ligands were eliminated from the protein and the polar hydrogen was added. The whole 6HGC was defined as a receptor and the site sphere was selected based on catalytic binding site of 6HGC. Compounds iBAP and iBAP-II were placed during the molecular docking procedure. Interactions of the docked protein with ligand were analyzed after the end of molecular docking.

### RNA interference and real-time PCR

The cells were infected with lentivirus containing short-hairpin RNAs (shRNAs) in the presence of 4 μg/ml Polybrene (Sigma) for 24 h in RPMI-1640 supplemented with 10% FBS. The infected cells were selected with 2 μg/ml puromycin for an additional 48 h. The shRNA constructs were purchased from Sigma. The clone IDs for RNF2 are TRCN0000234588 (sh*RNF2*-1) and TRCN0000238779 (sh*RNF2*-2). The non-targeting (sh*Ctrl*) shRNA construct (SHC002) was purchased from Sigma. Primers for qPCR are as follows, forward: 5′- CAGCCACCCGAGATTGAGCA-3′, reverse: 5′-TAGTAGCGACGGGCGGTGTG-3′ (*18* *S*); forward: 5′-AGCTTCTCGACTTCACCAAC-3′, reverse: 5′-CAACGCCACTGACAAGAAAG-3′ (*ASCL1*); forward: 5′-GTGGATGACTGAGTACCTGAAC-3′, reverse: 5′-GCCAGGAGAAATCAAACAGAGG-3′ (*BCL2*); forward: 5′-GACAGATCACAGCTACGGTG-3′, reverse: 5′-GGCGGACTTCCTCAGAATTG-3′ (*MAD2L1*); forward: 5′-GACAGAAGCGAAATTAGTGCG-3′, reverse: 5′-TTGATGTCAGTTCAGGCTCC-3′ (C1QBP); forward: 5′-AGCAGAAACTTGACAGGCAG-3′, reverse: 5′-GAATTCTGTTTGCCCGACTTG-3′ (*GNL3*); forward: 5′-CACGGAGCCTGAAGATGAG-3′, reverse: 5′-TGGCAATGATATCTGAGTCGG-3′ (*SNRPA*);

forward: 5′-TGTGGGTGACGGTTTTGAG-3′, reverse: 5′-TCCTTGAAGAGACTTTCGGC-3′ (*SRM*); forward: 5′-ACTCACTTTGCCTCTCAGAAC-3′, reverse: 5′-CTTTCCTCTGCACCTTCTCG-3′ (*TFDP1*); forward: 5′-GATGCCACCTATACTTCTGCC-3′, reverse: 5′-TCCTTTGACATCTCCATTAGCC-3′ (*MCM7*); forward: 5′-ACAAGGGACCAAAGTAGCAG-3′, reverse: 5′-ATAAACGGAATCTGGAGGTGG-3′ (*SYNCRIP*); forward: 5′-CGTTCCAAGGCATCTGTGAG-3′, reverse: 5′-GATCAAATCCCCGCAATATTCC-3′ (*SNRPG*); forward: 5′-TGGAAATGACAAGGGTGGAC-3′, reverse: 5′-TCTTGATCCTCACGTTTCTGTG-3′ (*MRPL23*); forward: 5′-AACCTGGAATTGGAACGGAG-3′, reverse: 5′-GCATACCCTCTATTCTGACCG-3′ (*HNRNPR*); forward: 5′-AAGGAGAGCATCTTGGACTTG-3′, reverse: 5′-TCTCGCATGTACTCAATGGTG-3′ (LSM7); forward: 5′-CCTCTACGTTCAATGGCTTCC-3′, reverse: 5′-GCATCCTGTTCCTCTACTTCG-3′ (*ODC1*).

### RNA-seq

Paramagnetic beads coupled with oligo d(T) are combined with total RNA to isolate poly(A) + transcripts based on NEBNext^®^ Poly(A) mRNA Magnetic Isolation Module manual. Prior to first strand synthesis, samples were randomly primed (5′ d(N6) 3′ [N = A,C,G,T]) and fragmented based on manufacturer’s recommendations (NEBNext^®^ Ultra™ II RNA Nondirectional Library Prep Kit for Illumina^®^). The first strand is synthesized with the Protoscript II Reverse Transcriptase with a longer extension period (40 min for 42 °C). All remaining steps for library construction were used according to the NEBNext^®^ Ultra™ II RNA Nondirectional Library Prep Kit for Illumina^®^. Illumina 8-nt dual-indices were used. Samples were pooled and sequenced on a HiSeq with a read length configuration of 150 PE.

### RNA-seq analysis

Gene counts were computed by HTSeq and used as an input for edgeR 3.0.852. Genes with Benjamini-Hochburg adjusted *p*-values less than 0.01 were considered to be differentially expressed (unless otherwise specified). RNA-seq heatmaps adjacent to ChIP-seq heatmaps display log2 (fold change) values of genes corresponding to TSSs nearest to ChIP-seq peaks and were displayed using Java TreeView. GO functional analysis was carried out using GSEA and Metascape with default parameters.

### ChIP-seq assay

ChIP-seq was performed as previously described. Briefly, the cells were harvested and washed twice with ice-cold PBS, and then fixed with 1% paraformaldehyde for 10 min at RT. The cell pellets were washed twice with PBS after glycine quench, and the cell pellets were resuspended with lysis buffer 1 (50 mM HEPES, pH = 7.5, 140 mM NaCl, 1 mM EDTA, 10% Glycerol, 0.5% NP-40, 0.25% Triton X-100, and 1X protease inhibitors). The resuspended cells were then incubated on nutator at 4 °C for 10 min. The purified nuclei pellets were then centrifuged at 500 *g* for 5 min and the supernatant was discarded. The nuclei pellets were washed with lysis buffer 2 (10 mM Tris-HCl, pH = 8.0, 200 mM NaCl, 1 mM EDTA, 0.5 mM EGTA, and 1 X protease inhibitors) and resuspended with lysis buffer 3 (10 mM Tris-HCl, pH = 8.0, 1 mM EDTA, 0.1% SDS, and 1 X protease inhibitors). Sonication was performed with 1 ml Covaris tubes set to 10% duty factor, 175 peak intensity power, 200 cycles per burst for 60–600 s. 10% of 10X ChIP dilution buffer (10% Triton x-100, 1 M NaCl, 1% Na-Deoxycholate, 5% N-Lauroylsarcosine, and 5 mM EGTA) was added to the lysate and samples were centrifuged at maximum speed for 15 min at 4 °C to pellet debris. For immunoprecipitation, 5 µg of antibody was added to each sample. After incubation at 4 °C on nutator overnight, 80 µl Protein A/G Agarose beads were added for each sample for 4 h. The agarose beads were washed 4 times with RIPA buffer (50 mM HEPES, pH = 7.5, 500 mM LiCl, 1 mM EDTA, 1.0% NP-40, and 0.7% Na-Deoxycholate), followed by once with ice-cold TE buffer (with 50 mM NaCl). The binding DNA was eluted with elution buffer (50 mM Tris-HCl, pH = 8.0, 10 mM EDTA, and 1.0% SDS) and then reverse cross-linked using a 65 °C oven for 6–15 h, followed by protease K digestion at 55 °C for 2 h.

### ChIP-seq analysis

For ChIP-seq analysis, all the peaks were processed with the MACS v1.4.2 software using default parameters and corresponding input samples. Metaplots and heatmaps were generated using ngsplot database to display ChIPseq signals. Peak annotation, motif analysis and super enhancer analysis were performed with HOMER. Correlation of BAP1, H2AUb, and RING1B ChIP-seq was analyzed with deepTools. Both TSS and non-TSS were clustered based on the peak annotation from HOMER.

### Animal experiments

All mouse work was performed following protocols approved by The Center for Comparative Medicine of Northwestern University. 5- to 6-week-old athymic nude mice were used for xenograft experiments. For tumor growth assays, 5.0 × 10^5^ KP1 or KP3 (mouse SCLC cell line) cells were inoculated into the right flank of nude mice. The mice were treated with either vehicle control (10% DMSO + 90% Cremophor) or iBAP-II (50 mg/kg/day). Tumor growth was monitored every 4 or 5 days 2 weeks after inoculation.

### Statistical analyses

For statistical analyses, GraphPad Prism 7, Microsoft Excel, and R were used. All data involving a statistical analysis being reported that met the criteria to use the appropriate statistical tests; for the normal distribution of data, the empirical rule was used to infer the distribution. For growth curves and time-course, RNA-seq *t*-tests were calculated between the area-under-the-curve values. Statistical tests used are reported in the figure legends.

## Supplementary information

Supplementary Information

Supplementary Figure 1

Supplementary Figure 2

Supplementary Figure 3

Supplementary Figure 4

## Data Availability

NSG data generated for this study are available at the Gene Expression Omnibus (GEO) under accession number GSE191106.
