# Supplementary material for: Therapeutic targeting of BAP1/ASXL3 sub-complex in ASCL1-dependent small cell lung cancer
Source: Oncogene. 2022 Feb 22;41(15):2152–62. doi: 10.1038/s41388-022-02240-x (PMC8993689; doi:10.1038/s41388-022-02240-x)
Supplement: Supplementary file 1 — Supplementary Information [file 41388_2022_2240_MOESM1_ESM.docx]

**Supplementary Information**

**Supplementary Method**

**General procedure for the synthesis of racemic 4-phenyl-3a,4,5,9b-tetrahydro-3H-cyclopenta[c]quinoline derivatives.**

A mixture of substituted benzaldehyde (10 mmol) and substituted aniline (11 mmol) in MeCN (20 mL) was stirred at 0^o^C for 0.5 h. TFA (1.3 mL, 10 mmol) and cyclopentadiene (1.3 mL, 15 mmol) were added and the reaction was stirred at 0 °C for 1 h. Upon completion, saturated NaHCO3 (20 mL) was added and extracted with EtOAc (3 × 20 mL). The combined organic phases were washed with saturated brine (20 mL), dried with anhydrous Na2SO4, filtered, and concentrated in vacuo. The residue was purified by column chromatography (petroleum ether/EtOAc 95:5) to provide the tetrahydroquinolines.

**4-Phenyl-3a,4,5,9b-tetrahydro-3*H*-cyclopenta[*c*]quinoline (1)**

Brown solid, yield: 50%. ^1^H NMR (300 MHz, DMSO-*d*_6_) δ 7.53 – 7.25 (m, 5H), 7.09 – 6.97 (m, 1H), 6.90 (td, *J* = 7.5, 1.5 Hz, 1H), 6.77 (dd, *J* = 8.0, 1.4 Hz, 1H), 6.62 (td, *J* = 7.3, 1.4 Hz, 1H), 5.92 – 5.84 (m, 1H), 5.65 – 5.54 (m, 2H), 4.55 (d, *J* = 3.2 Hz, 1H), 4.06 (d, *J* = 8.6 Hz, 1H), 3.06 – 2.84 (m, 1H), 2.51 – 2.38 (m, 1H), 1.64 (ddt, *J* = 16.2, 8.9, 2.1 Hz, 1H). HRMS (ESI): *m/z* calcd C_18_H_18_N (M + H^+^) = 248.1439, found = 248.1436.

6-Methyl-4-phenyl-3a,4,5,9b-tetrahydro-3*H*-cyclopenta[*c*]quinoline (2)

Orange solid, yield: 58%. ^1^H NMR (300 MHz, DMSO-*d*_6_) δ 7.57 – 7.50 (m, 2H), 7.47 – 7.27 (m, 3H), 6.92 (d, *J* = 7.6 Hz, 1H), 6.86 – 6.81 (m, 1H), 6.60 (td, *J* = 7.4, 1.3 Hz, 1H), 5.94 – 5.84 (m, 1H), 5.63 – 5.53 (m, 1H), 4.60 (d, *J* = 3.3 Hz, 1H), 4.50 (s, 1H), 4.10 (d, *J* = 8.8 Hz, 1H), 3.04 – 2.87 (m, 1H), 2.56 – 2.39 (m, 1H), 2.17 (s, 3H), 1.72 – 1.57 (m, 1H). HRMS (ESI): *m/z* calcd C_19_H_20_N (M + H^+^) = 262.1596, found = 262.1595.

6-Nitro-4-phenyl-3a,4,5,9b-tetrahydro-3*H*-cyclopenta[*c*]quinoline (3)

Orange solid, yield: 62%. ^1^H NMR (300 MHz, DMSO-*d*_6_) δ 7.96 – 7.85 (m, 2H), 7.51 – 7.42 (m, 5H), 7.40 – 7.31 (m, 1H), 6.77 (ddd, *J* = 8.3, 7.3, 0.7 Hz, 1H), 5.91 (dd, *J* = 4.7, 2.5 Hz, 1H), 5.63 (d, *J* = 5.5 Hz, 1H), 4.87 (d, *J* = 3.6 Hz, 1H), 4.22 (d, *J* = 9.0 Hz, 1H), 3.08 (dt, *J* = 10.4, 5.3 Hz, 1H), 2.40 (ddd, *J* = 16.5, 9.6, 2.4 Hz, 1H), 1.80 – 1.65 (m, 1H). HRMS (ESI): *m/z* calcd C_18_H_17_N_2_O_2_ (M + H^+^) = 293.1290, found = 293.1290.

7-Nitro-4-phenyl-3a,4,5,9b-tetrahydro-3*H*-cyclopenta[*c*]quinoline (4)

Orange solid, yield: 42%. ^1^H NMR (300 MHz, DMSO-*d*_6_) δ 7.50 – 7.24 (m, 5H), 7.19 – 7.02 (m, 3H), 6.41 (s, 1H), 5.73 – 5.62 (m, 1H), 5.52 – 5.42 (m, 1H), 4.65 – 4.54 (m, 2H), 3.04 (q, *J* = 9.3 Hz, 1H), 2.50 – 2.38 (m, 1H), 1.74 – 1.57 (m, 1H). HRMS (ESI): *m/z* calcd C_18_H_17_N_2_O_2_ (M + H^+^) = 293.1290, found = 293.1294.

8-Nitro-4-phenyl-3a,4,5,9b-tetrahydro-3*H*-cyclopenta[*c*]quinoline (5, iBAP)

Yellow solid, yield: 90%. ^1^H NMR (300 MHz, DMSO-*d*_6_) δ 7.92 (d, *J* = 2.6 Hz, 1H), 7.83 (dd, *J* = 9.0, 2.7 Hz, 1H), 7.48 – 7.25 (m, 6H), 6.84 (d, *J* = 9.0 Hz, 1H), 6.03 (ddt, *J* = 5.9, 3.1, 1.5 Hz, 1H), 5.66 – 5.58 (m, 1H), 4.76 (d, *J* = 3.5 Hz, 1H), 4.10 (d, *J* = 8.0 Hz, 1H), 2.94 (dddd, *J* = 11.9, 9.9, 5.3, 2.3 Hz, 1H), 2.34 (ddq, *J* = 16.6, 10.0, 2.2 Hz, 1H), 1.63 (ddt, *J* = 16.2, 8.6, 2.4 Hz, 1H). HRMS (ESI): *m/z* calcd C_18_H_17_N_2_O_2_ (M + H^+^) = 293.1290, found = 293.1285.

7,8,9-Trimethoxy-4-phenyl-3a,4,5,9b-tetrahydro-3*H*-cyclopenta[*c*]quinoline (6)

White solid, yield: 38%. ^1^H NMR (300 MHz, DMSO-*d*_6_) δ 7.49 – 7.35 (m, 4H), 7.29 (t, *J* = 7.1 Hz, 1H), 6.29 (s, 1H), 5.86 (s, 1H), 5.55 (d, *J* = 5.6 Hz, 1H), 5.45 (s, 1H), 4.44 (d, *J* = 3.1 Hz, 1H), 4.02 (d, *J* = 9.3 Hz, 1H), 3.85 (s, 3H), 3.70 (s, 3H), 3.65 (s, 3H), 3.03 – 2.88 (m, 1H), 2.51 – 2.40 (m, 1H), 1.62 (dd, *J* = 16.2, 8.7 Hz, 1H). HRMS (ESI): *m/z* calcd C_21_H_24_NO_3_ (M + H^+^) = 338.1756, found = 338.1763.

8-Fluoro-4-phenyl-3a,4,5,9b-tetrahydro-3*H*-cyclopenta[*c*]quinoline (7)

Pale purple solid, yield: 40%. ^1^H NMR (300 MHz, DMSO-*d*_6_) δ 7.53 – 7.25 (m, 5H), 6.94 – 6.84 (m, 1H), 6.76 (dd, *J* = 7.4, 1.9 Hz, 2H), 5.88 (dq, *J* = 5.5, 2.0 Hz, 1H), 5.62 (d, *J* = 4.6 Hz, 2H), 4.51 (d, *J* = 3.2 Hz, 1H), 4.05 (d, *J* = 8.9 Hz, 1H), 3.02 – 2.87 (m, 1H), 2.45 (ddt, *J* = 14.2, 8.0, 2.4 Hz, 1H), 1.64 (ddd, *J* = 11.2, 9.0, 4.7 Hz, 1H). HRMS (ESI): *m/z* calcd C_18_H_17_FN (M + H^+^) = 266.1345, found = 266.1343.

8-Chloro-4-phenyl-3a,4,5,9b-tetrahydro-3*H*-cyclopenta[*c*]quinoline (8)

Purple solid, yield: 42%. ^1^H NMR (300 MHz, DMSO-*d*_6_) δ 7.52 – 7.22 (m, 5H), 7.08 (d, *J* = 2.4 Hz, 1H), 6.92 (td, *J* = 8.6, 2.3 Hz, 1H), 6.79 (d, *J* = 8.6 Hz, 1H), 5.97 – 5.84 (m, 1H), 5.62 (d, *J* = 5.6 Hz, 1H), 4.55 (d, *J* = 3.2 Hz, 1H), 4.05 (d, *J* = 8.8 Hz, 2H), 2.94 (qd, *J* = 8.9, 3.3 Hz, 1H), 2.44 (ddd, *J* = 11.8, 9.5, 4.7 Hz, 1H), 1.65 (ddt, *J* = 16.1, 8.7, 2.1 Hz, 1H). HRMS (ESI): *m/z* calcd C_18_H_17_ClN (M + H^+^) = 282.1050, found = 282.1056.

8-Bromo-4-phenyl-3a,4,5,9b-tetrahydro-3*H*-cyclopenta[*c*]quinoline (9)

Brown solid, yield: 43%. ^1^H NMR (300 MHz, DMSO-*d*_6_) δ 7.54 – 7.25 (m, 5H), 7.20 (d, *J* = 2.4 Hz, 1H), 7.05 (dd, *J* = 8.5, 2.4 Hz, 1H), 6.73 (d, *J* = 8.6 Hz, 1H), 6.00 – 5.84 (m, 2H), 5.70 – 5.55 (m, 1H), 4.55 (d, *J* = 3.3 Hz, 1H), 4.05 (d, *J* = 8.8 Hz, 1H), 3.03 – 2.84 (m, 1H), 2.51 – 2.37 (m, 1H), 1.77 – 1.56 (m, 1H). HRMS (ESI): *m/z* calcd C_18_H_17_BrN (M + H^+^) = 326.0544, found = 326.0545.

4-Phenyl-3a,4,5,9b-tetrahydro-3*H*-cyclopenta[*c*]quinoline-8-carbonitrile (11)

Pale purple solid, yield: 67%. ^1^H NMR (300 MHz, DMSO-*d*_6_) δ 7.51 – 7.39 (m, 5H), 7.39 – 7.26 (m, 2H), 6.85 (d, *J* = 8.5 Hz, 1H), 6.70 (s, 1H), 6.03 – 5.94 (m, 1H), 5.62 (d, *J* = 5.6 Hz, 1H), 4.69 (d, *J* = 3.4 Hz, 1H), 4.06 (d, *J* = 8.7 Hz, 1H), 3.02 – 2.87 (m, 1H), 2.38 (ddd, *J* = 15.7, 9.8, 2.4 Hz, 1H), 1.72 – 1.58 (m, 1H). HRMS (ESI): *m/z* calcd C_19_H_17_N_2_ (M + H^+^) = 273.1392, found = 273.1390.

4-Phenyl-3a,4,5,9b-tetrahydro-3*H*-cyclopenta[*c*]quinoline-8-carboxylic acid (12)

White solid, yield: 85%. ^1^H NMR (300 MHz, DMSO-*d*_6_) δ 12.18 (s, 1H), 7.62 (d, *J* = 1.9 Hz, 1H), 7.56 – 7.27 (m, 6H), 6.79 (d, *J* = 8.4 Hz, 1H), 6.46 (s, 1H), 5.94 (dt, *J* = 5.6, 2.7 Hz, 1H), 5.62 (d, *J* = 5.6 Hz, 1H), 4.67 (d, *J* = 3.3 Hz, 1H), 4.09 (d, *J* = 8.7 Hz, 1H), 3.03 – 2.87 (m, 1H), 2.42 (ddt, *J* = 16.4, 9.5, 2.3 Hz, 1H), 1.72 – 1.57 (m, 1H). HRMS (ESI): *m/z* calcd C_19_H_18_NO_2_ (M + H^+^) = 292.1338, found = 273.1338.

8-(Benzyloxy)-4-phenyl-3a,4,5,9b-tetrahydro-3*H*-cyclopenta[*c*]quinoline (13)

White solid, yield: 63%. ^1^H NMR (300 MHz, DMSO-*d*_6_) δ 7.82 – 7.66 (m, 2H), 7.55 – 7.25 (m, 9H), 7.13 (d, *J* = 2.7 Hz, 1H), 6.97 (dd, *J* = 8.8, 2.8 Hz, 1H), 6.00 (dd, *J* = 5.5, 2.6 Hz, 1H), 5.71 (d, *J* = 5.6 Hz, 1H), 5.16 (s, 2H), 4.89 (d, *J* = 3.4 Hz, 1H), 4.32 – 4.15 (m, 2H), 3.10 (qd, *J* = 9.3, 3.4 Hz, 1H), 2.91 – 2.75 (m, 1H), 1.95 (td, *J* = 11.6, 10.6, 4.8 Hz, 1H). HRMS (ESI): *m/z* calcd C_25_H_24_NO (M + H^+^) = 354.1858, found = 354.1852.

4-Phenyl-3a,4,5,9b-tetrahydro-3*H*-cyclopenta[*c*]quinolin-8-ol (14)

Pale pink solid, yield: 50%. ^1^H NMR (300 MHz, DMSO-*d*_6_) δ 9.08 (s, 1H), 7.68 – 7.28 (m, 6H), 6.95 (d, *J* = 8.0 Hz, 1H), 6.69 (s, 1H), 6.64 – 6.56 (m, 1H), 5.89 (dd, *J* = 5.8, 2.7 Hz, 1H), 5.72 – 5.64 (m, 1H), 4.75 (s, 1H), 4.11 (d, *J* = 9.1 Hz, 1H), 3.05 (qd, *J* = 9.2, 3.3 Hz, 1H), 2.66 – 2.55 (m, 1H), 1.85 (dd, *J* = 17.0, 9.6 Hz, 1H). HRMS (ESI): *m/z* calcd C_18_H_18_NO (M + H^+^) = 264.1388, found = 264.1395.

8-Methoxy-4-phenyl-3a,4,5,9b-tetrahydro-3*H*-cyclopenta[*c*]quinoline (15)

Dark purple solid, yield: 39%. ^1^H NMR (300 MHz, DMSO-*d*_6_) δ 7.58 – 7.30 (m, 5H), 6.96 (d, *J* = 8.7 Hz, 1H), 6.84 (d, *J* = 2.8 Hz, 1H), 6.72 (dd, *J* = 8.7, 2.9 Hz, 1H), 5.95 (ddq, *J* = 5.8, 2.8, 1.4 Hz, 1H), 5.71 – 5.60 (m, 1H), 4.68 (d, *J* = 3.3 Hz, 2H), 4.19 – 4.08 (m, 1H), 3.74 (s, 3H), 3.03 (qd, *J* = 9.1, 3.4 Hz, 1H), 2.62 – 2.55 (m, 1H), 1.92 – 1.68 (m, 1H). HRMS (ESI): *m/z* calcd C_19_H_20_NO (M + H^+^) = 278.1545, found = 278.1541.

4-(2-Fluorophenyl)-8-nitro-3a,4,5,9b-tetrahydro-3*H*-cyclopenta[*c*]quinoline (17)

Pale brown solid, yield: 52%. ^1^H NMR (300 MHz, DMSO-*d*_6_) δ 7.95 (d, *J* = 2.6 Hz, 1H), 7.87 (dd, *J* = 9.0, 2.7 Hz, 1H), 7.60 (td, *J* = 7.6, 1.8 Hz, 1H), 7.48 – 7.20 (m, 4H), 6.87 (d, *J* = 9.0 Hz, 1H), 6.05 (dt, *J* = 5.8, 2.6 Hz, 1H), 5.64 (d, *J* = 5.6 Hz, 1H), 5.05 (d, *J* = 3.5 Hz, 1H), 4.15 (d, *J* = 8.6 Hz, 1H), 3.02 (dtd, *J* = 12.2, 9.2, 3.1 Hz, 1H), 2.37 (ddq, *J* = 16.6, 9.9, 2.3 Hz, 1H), 1.77 – 1.62 (m, 1H). HRMS (ESI): *m/z* calcd C_18_H_16_FN_2_O_2_ (M + H^+^) = 311.1196, found = 311.1196.

4-(2-Bromophenyl)-8-nitro-3a,4,5,9b-tetrahydro-3*H*-cyclopenta[*c*]quinoline (18)

Pale brown solid, yield: 64%. ^1^H NMR (300 MHz, DMSO-*d*_6_) δ 7.97 (dd, *J* = 2.7, 1.0 Hz, 1H), 7.88 (dd, *J* = 9.0, 2.7 Hz, 1H), 7.71 (dd, *J* = 8.0, 1.3 Hz, 1H), 7.63 (dd, *J* = 7.8, 1.8 Hz, 1H), 7.52 (td, *J* = 7.5, 1.3 Hz, 1H), 7.38 – 7.23 (m, 2H), 6.86 (d, *J* = 9.0 Hz, 1H), 6.16 – 5.99 (m, 1H), 5.67 (d, *J* = 5.6 Hz, 1H), 5.03 (d, *J* = 3.5 Hz, 1H), 4.16 (d, *J* = 8.6 Hz, 1H), 3.14 (qd, *J* = 8.8, 3.4 Hz, 1H), 2.50 – 2.29 (m, 1H), 1.72 – 1.52 (m, 1H). HRMS (ESI): *m/z* calcd C_18_H_16_BrN_2_O_2_ (M + H^+^) = 371.0395, found = 371.0393.

4-(2-Iodophenyl)-8-nitro-3a,4,5,9b-tetrahydro-3*H*-cyclopenta[*c*]quinoline (19)

Pale brown solid, yield: 97%. ^1^H NMR (300 MHz, DMSO-*d*_6_) δ 8.06 – 7.77 (m, 3H), 7.61 – 7.43 (m, 2H), 7.29 (s, 1H), 7.14 (ddd, *J* = 7.8, 5.8, 3.2 Hz, 1H), 6.85 (d, *J* = 9.0 Hz, 1H), 6.08 (t, *J* = 3.9 Hz, 1H), 5.67 (d, *J* = 5.6 Hz, 1H), 4.86 (d, *J* = 3.5 Hz, 1H), 4.15 (d, *J* = 8.7 Hz, 1H), 3.21 – 3.02 (m, 1H), 2.49 – 2.30 (m, 1H), 1.59 (dd, *J* = 15.6, 8.6 Hz, 1H). HRMS (ESI): *m/z* calcd C_18_H_16_IN_2_O_2_ (M + H^+^) = 419.0256, found = 419.0261.

8-Nitro-4-(2-nitrophenyl)-3a,4,5,9b-tetrahydro-3*H*-cyclopenta[*c*]quinoline (20)

Pale brown solid, yield: 75%. ^1^H NMR (300 MHz, DMSO-*d*_6_) δ 8.09 – 7.77 (m, 5H), 7.72 – 7.58 (m, 1H), 7.25 (s, 1H), 6.83 (d, *J* = 9.0 Hz, 1H), 6.09 (dt, *J* = 6.0, 2.6 Hz, 1H), 5.69 (d, *J* = 5.7 Hz, 1H), 5.12 (d, *J* = 3.3 Hz, 1H), 4.14 (d, *J* = 8.6 Hz, 1H), 3.23 – 3.04 (m, 1H), 2.52 – 2.41 (m, 1H), 1.73 (dt, *J* = 15.0, 9.6 Hz, 1H). HRMS (ESI): *m/z* calcd C_18_H_16_N_3_O_4_ (M + H^+^) = 338.1141, found = 338.1135.

2-(8-Nitro-3a,4,5,9b-tetrahydro-3*H*-cyclopenta[*c*]quinolin-4-yl)benzonitrile (21)

Pale yellow solid, yield: 46%. ^1^H NMR (300 MHz, DMSO-*d*_6_) δ 8.03 – 7.74 (m, 5H), 7.58 (td, *J* = 7.4, 1.5 Hz, 1H), 7.42 – 7.30 (m, 1H), 6.87 (d, *J* = 9.0 Hz, 1H), 6.15 – 6.04 (m, 1H), 5.67 (d, *J* = 5.7 Hz, 1H), 5.08 (d, *J* = 3.7 Hz, 1H), 4.20 (d, *J* = 8.6 Hz, 1H), 3.17 – 2.99 (m, 1H), 2.48 – 2.31 (m, 1H), 1.66 (dd, *J* = 15.7, 8.4 Hz, 1H). HRMS (ESI): *m/z* calcd C_19_H_16_N_3_O_2_ (M + H^+^) = 318.1243, found = 318.1242.

8-Nitro-4-(2-(trifluoromethyl)phenyl)-3a,4,5,9b-tetrahydro-3*H*-cyclopenta[*c*]quinoline (22)

Yellow solid, yield: 74%. ^1^H NMR (300 MHz, DMSO-*d*_6_) δ 8.00 – 7.74 (m, 5H), 7.61 (t, *J* = 7.6 Hz, 1H), 7.39 (s, 1H), 6.82 (d, *J* = 9.0 Hz, 1H), 6.12 (dt, *J* = 5.7, 2.7 Hz, 1H), 5.70 (d, *J* = 5.6 Hz, 1H), 5.05 (d, *J* = 3.2 Hz, 1H), 4.15 (d, *J* = 7.5 Hz, 1H), 3.02 – 2.74 (m, 1H), 2.67 – 2.55 (m, 1H), 1.79 – 1.63 (m, 1H). HRMS (ESI): *m/z* calcd C_19_H_16_F_3_N_2_O_2_ (M + H^+^) = 361.1164, found = 361.1172.

4-(2,3-Dichlorophenyl)-8-nitro-3a,4,5,9b-tetrahydro-3*H*-cyclopenta[*c*]quinoline (23)

Yellow solid, yield: 98%. ^1^H NMR (300 MHz, DMSO-*d*_6_) δ 7.97 (d, *J* = 2.6 Hz, 1H), 7.88 (dd, *J* = 9.0, 2.6 Hz, 1H), 7.72 – 7.59 (m, 2H), 7.50 (t, *J* = 7.9 Hz, 1H), 7.24 (s, 1H), 6.86 (d, *J* = 9.0 Hz, 1H), 6.07 (s, 1H), 5.66 (d, *J* = 5.6 Hz, 1H), 5.12 (d, *J* = 3.4 Hz, 1H), 4.17 (d, *J* = 8.7 Hz, 1H), 3.23 – 3.04 (m, 1H), 2.44 – 2.26 (m, 1H), 1.63 (dd, *J* = 16.0, 8.4 Hz, 1H). HRMS (ESI): *m/z* calcd C_18_H_15_Cl_2_N_2_O_2_ (M + H^+^) = 361.0511, found = 361.0514.

4-(2-Methoxyphenyl)-8-nitro-3a,4,5,9b-tetrahydro-3*H*-cyclopenta[*c*]quinoline (24)

Yellow solid, yield: 85%. ^1^H NMR (300 MHz, DMSO-*d*_6_) δ 7.97 – 7.90 (m, 1H), 7.85 (dd, *J* = 9.0, 2.7 Hz, 1H), 7.46 (dd, *J* = 7.5, 1.8 Hz, 1H), 7.40 – 7.28 (m, 1H), 7.15 – 7.00 (m, 3H), 6.85 (d, *J* = 9.0 Hz, 1H), 6.07 – 5.99 (m, 1H), 5.64 (d, *J* = 5.6 Hz, 1H), 5.03 (d, *J* = 3.4 Hz, 1H), 4.11 (d, *J* = 8.7 Hz, 1H), 3.86 (s, 3H), 3.12 – 3.03 (m, 1H), 2.41 – 2.26 (m, 1H), 1.62 (dd, *J* = 15.7, 8.6 Hz, 1H). HRMS (ESI): *m/z* calcd C_19_H_19_N_2_O_3_ (M + H^+^) = 323.1396, found = 323.1392.

4-(3-Fluorophenyl)-8-nitro-3a,4,5,9b-tetrahydro-3H-cyclopenta[c]quinoline (25)

Yellow solid, yield: 54%. ^1^H NMR (300 MHz, DMSO-*d*_6_) δ 7.99 – 7.92 (m, 1H), 7.87 (dd, *J* = 9.0, 2.7 Hz, 1H), 7.55 – 7.42 (m, 1H), 7.37 – 7.25 (m, 3H), 7.24 – 7.11 (m, 1H), 6.86 (d, *J* = 9.0 Hz, 1H), 6.11 – 6.01 (m, 1H), 5.65 (d, *J* = 5.6 Hz, 1H), 4.83 (d, *J* = 3.6 Hz, 1H), 4.13 (d, *J* = 8.6 Hz, 1H), 3.00 (dt, *J* = 9.9, 4.6 Hz, 1H), 2.41 – 2.26 (m, 1H), 1.67 (dd, *J* = 15.7, 8.4 Hz, 1H). HRMS (ESI): *m/z* calcd C_18_H_16_FN_2_O_2_ (M + H^+^) = 311.1196, found = 311.1200.

4-(3-Chlorophenyl)-8-nitro-3a,4,5,9b-tetrahydro-3*H*-cyclopenta[*c*]quinoline (26)

Yellow solid, yield: 65%. ^1^H NMR (300 MHz, DMSO-*d*_6_) δ 7.95 (d, *J* = 2.6 Hz, 1H), 7.87 (dd, *J* = 9.0, 2.7 Hz, 1H), 7.58 – 7.36 (m, 4H), 7.32 (s, 1H), 6.86 (d, *J* = 9.0 Hz, 1H), 6.10 – 6.01 (m, 1H), 5.64 (d, *J* = 5.6 Hz, 1H), 4.82 (d, *J* = 3.5 Hz, 1H), 4.12 (d, *J* = 8.6 Hz, 1H), 3.08 – 2.91 (m, 1H), 2.32 (ddq, *J* = 16.8, 10.1, 2.3 Hz, 1H), 1.67 (ddt, *J* = 16.1, 8.5, 2.5 Hz, 1H). HRMS (ESI): *m/z* calcd C_18_H_16_ClN_2_O_2_ (M + H^+^) = 327.0900, found = 327.0898.

4-(3-Bromophenyl)-8-nitro-3a,4,5,9b-tetrahydro-3*H*-cyclopenta[*c*]quinoline (27)

Pale brown solid, yield: 91%. ^1^H NMR (300 MHz, DMSO-*d*_6_) δ 7.95 (d, *J* = 2.6 Hz, 1H), 7.86 (dd, *J* = 9.0, 2.7 Hz, 1H), 7.65 (d, *J* = 1.8 Hz, 1H), 7.59 – 7.25 (m, 4H), 6.87 (d, *J* = 9.1 Hz, 1H), 6.10 – 5.98 (m, 1H), 5.64 (d, *J* = 5.6 Hz, 1H), 4.79 (d, *J* = 3.5 Hz, 1H), 4.11 (d, *J* = 8.5 Hz, 1H), 2.98 (qd, *J* = 9.1, 3.3 Hz, 1H), 2.32 (ddt, *J* = 16.0, 9.7, 2.3 Hz, 1H), 1.66 (ddd, *J* = 17.3, 8.4, 2.7 Hz, 1H). HRMS (ESI): *m/z* calcd C_18_H_16_BrN_2_O_2_ (M + H^+^) = 371.0395, found = 371.0394.

4-(3-Iodophenyl)-8-nitro-3a,4,5,9b-tetrahydro-3*H*-cyclopenta[*c*]quinoline (28)

Pale brown solid, yield: 77%. ^1^H NMR (300 MHz, DMSO-*d*_6_) δ 7.95 (d, *J* = 2.6 Hz, 1H), 7.92 – 7.80 (m, 2H), 7.72 (dt, *J* = 8.1, 1.3 Hz, 1H), 7.50 (d, *J* = 7.7 Hz, 1H), 7.33 – 7.20 (m, 2H), 6.86 (d, *J* = 9.0 Hz, 1H), 6.10 – 6.01 (m, 1H), 5.70 – 5.61 (m, 1H), 4.78 (d, *J* = 3.5 Hz, 1H), 4.12 (d, *J* = 8.6 Hz, 1H), 3.02 – 2.93 (m, 1H), 2.38 – 2.24 (m, 1H), 1.67 (dd, *J* = 15.8, 8.5 Hz, 1H). HRMS (ESI): *m/z* calcd C_18_H_16_IN_2_O_2_ (M + H^+^) = 419.0256, found = 419.0253.

8-Nitro-4-(3-nitrophenyl)-3a,4,5,9b-tetrahydro-3*H*-cyclopenta[*c*]quinolin (29)

Pale brown solid, yield: 80%. ^1^H NMR (300 MHz, DMSO-*d*_6_) δ 8.34 (t, *J* = 2.0 Hz, 1H), 8.23 (dd, *J* = 8.2, 2.3 Hz, 1H), 8.02 – 7.84 (m, 3H), 7.75 (t, *J* = 7.9 Hz, 1H), 7.42 (s, 1H), 6.90 (d, *J* = 8.9 Hz, 1H), 6.08 (dt, *J* = 5.7, 2.8 Hz, 1H), 5.64 (d, *J* = 5.7 Hz, 1H), 4.99 (d, *J* = 3.6 Hz, 1H), 4.16 (d, *J* = 8.5 Hz, 1H), 3.06 (qd, *J* = 9.2, 3.4 Hz, 1H), 2.40 – 2.24 (m, 1H), 1.65 (ddd, *J* = 16.4, 8.2, 2.8 Hz, 1H). HRMS (ESI): *m/z* calcd C_18_H_16_N_3_O_4_ (M + H^+^) = 338.1141, found = 338.1143.

3-(8-Nitro-3a,4,5,9b-tetrahydro-3*H*-cyclopenta[*c*]quinolin-4-yl)benzonitrile (30)

Dark brown solid, yield: 80%. ^1^H NMR (300 MHz, DMSO-*d*_6_) δ 8.03 – 7.77 (m, 5H), 7.74 – 7.57 (m, 1H), 7.33 (s, 1H), 6.86 (d, *J* = 9.0 Hz, 1H), 6.13 – 5.98 (m, 1H), 5.70 – 5.57 (m, 1H), 4.88 (d, *J* = 3.5 Hz, 1H), 4.24 – 4.06 (m, 1H), 3.12 – 2.95 (m, 1H), 2.43 – 2.23 (m, 1H), 1.64 (dd, *J* = 15.6, 8.5 Hz, 1H). HRMS (ESI): *m/z* calcd C_19_H_16_N_3_O_2_ (M + H^+^) = 318.1243, found = 318.1239.

8-Nitro-4-(3-(trifluoromethyl)phenyl)-3a,4,5,9b-tetrahydro-3*H*-cyclopenta[*c*]quinoline (31)

Yellow solid, yield: 45%. ^1^H NMR (300 MHz, DMSO-*d*_6_) δ 8.02 – 7.95 (m, 1H), 7.89 (dd, *J* = 9.0, 2.7 Hz, 1H), 7.84 – 7.65 (m, 4H), 7.37 (s, 1H), 6.89 (d, *J* = 9.0 Hz, 1H), 6.08 (dq, *J* = 6.6, 3.0, 2.5 Hz, 1H), 5.70 – 5.62 (m, 1H), 4.94 (d, *J* = 3.5 Hz, 1H), 4.16 (d, *J* = 8.6 Hz, 1H), 3.08 – 2.97 (m, 1H), 2.46 – 2.21 (m, 1H), 1.72 – 1.58 (m, 1H). HRMS (ESI): *m/z* calcd C_19_H_16_F_3_N_2_O_2_ (M + H^+^) = 361.1164, found = 361.1162.

3-(8-Nitro-3a,4,5,9b-tetrahydro-3*H*-cyclopenta[*c*]quinolin-4-yl)phenol (32)

Yellow solid, yield: 85%. ^1^H NMR (300 MHz, DMSO-*d*_6_) δ 9.53 (s, 1H), 7.93 (d, *J* = 2.8 Hz, 1H), 7.85 (dd, *J* = 9.0, 2.7 Hz, 1H), 7.31 – 7.18 (m, 2H), 6.93 – 6.82 (m, 3H), 6.74 (ddd, *J* = 8.1, 2.4, 1.0 Hz, 1H), 6.10 – 6.00 (m, 1H), 5.70 – 5.62 (m, 1H), 4.70 (d, *J* = 3.5 Hz, 1H), 4.12 (d, *J* = 8.6 Hz, 1H), 2.99 – 2.85 (m, 1H), 2.35 (ddd, *J* = 16.5, 9.9, 2.3 Hz, 1H), 1.72 (ddd, *J* = 15.5, 8.7, 4.4 Hz, 1H). HRMS (ESI): *m/z* calcd C_18_H_17_N_2_O_3_ (M + H^+^) = 309.1239, found = 309.1236.

8-Nitro-4-(m-tolyl)-3a,4,5,9b-tetrahydro-3*H*-cyclopenta[*c*]quinoline (33)

Yellow solid, yield: 55%. ^1^H NMR (300 MHz, DMSO-*d*_6_) δ 7.93 (d, *J* = 2.6 Hz, 1H), 7.85 (dd, *J* = 9.0, 2.6 Hz, 1H), 7.36 – 7.21 (m, 4H), 7.15 (d, *J* = 7.2 Hz, 1H), 6.86 (d, *J* = 9.0 Hz, 1H), 6.04 (ddt, *J* = 5.9, 2.9, 1.6 Hz, 1H), 5.64 (d, *J* = 5.7 Hz, 1H), 4.73 (d, *J* = 3.5 Hz, 1H), 4.21 – 4.02 (m, 1H), 2.94 (dq, *J* = 8.0, 5.8 Hz, 1H), 2.46 – 2.26 (m, 4H), 1.79 – 1.55 (m, 1H). HRMS (ESI): *m/z* calcd C_19_H_19_N_2_O_2_ (M + H^+^) = 307.1447, found = 307.1450.

4-(3-Methoxyphenyl)-8-nitro-3a,4,5,9b-tetrahydro-3*H*-cyclopenta[*c*]quinoline (34)

Pale yellow solid, yield: 52%. ^1^H NMR (300 MHz, DMSO-*d*_6_) δ 7.94 (d, *J* = 2.6 Hz, 1H), 7.85 (dd, *J* = 9.0, 2.7 Hz, 1H), 7.40 – 7.25 (m, 2H), 7.09 – 6.99 (m, 2H), 6.96 – 6.81 (m, 2H), 6.05 (dq, *J* = 5.7, 2.0 Hz, 1H), 5.65 (d, *J* = 5.7 Hz, 1H), 4.76 (d, *J* = 3.5 Hz, 1H), 4.12 (d, *J* = 8.6 Hz, 1H), 3.80 (s, 3H), 3.03 – 2.91 (m, 1H), 2.42 – 2.28 (m, 1H), 1.68 (dd, *J* = 15.8, 8.4 Hz, 1H). HRMS (ESI): *m/z* calcd C_19_H_19_N_2_O_3_ (M + H^+^) = 323.1396, found = 323.1395.

4-(3,4-Dimethoxyphenyl)-8-nitro-3a,4,5,9b-tetrahydro-3*H*-cyclopenta[*c*]quinoline (35)

Pale yellow solid, yield: 37%. ^1^H NMR (300 MHz, DMSO-*d*_6_) δ 7.94 (d, *J* = 2.7 Hz, 1H), 7.85 (dd, *J* = 9.0, 2.7 Hz, 1H), 7.26 (s, 1H), 7.07 – 6.93 (m, 3H), 6.85 (d, *J* = 9.0 Hz, 1H), 6.11 – 6.01 (m, 1H), 5.66 (d, *J* = 5.6 Hz, 1H), 4.72 (d, *J* = 3.5 Hz, 1H), 4.11 (d, *J* = 8.6 Hz, 1H), 3.81 (s, 3H), 3.79 (s, 3H), 3.05 – 2.87 (m, 1H), 2.46 – 2.31 (m, 1H), 1.83 – 1.62 (m, 1H). HRMS (ESI): *m/z* calcd C_20_H_21_N_2_O_4_ (M + H^+^) = 353.1501, found = 353.1494.

4-(4-Fluorophenyl)-8-nitro-3a,4,5,9b-tetrahydro-3*H*-cyclopenta[*c*]quinoline (36)

Yellow solid, yield: 63%. ^1^H NMR (300 MHz, DMSO-*d*_6_) δ 7.95 (d, *J* = 2.6 Hz, 1H), 7.86 (dd, *J* = 9.0, 2.7 Hz, 1H), 7.56 – 7.46 (m, 2H), 7.33 – 7.21 (m, 3H), 6.85 (d, *J* = 9.0 Hz, 1H), 6.06 (dq, *J* = 5.9, 3.0, 2.5 Hz, 1H), 5.65 (d, *J* = 5.6 Hz, 1H), 4.81 (d, *J* = 3.5 Hz, 1H), 4.12 (d, *J* = 8.6 Hz, 1H), 3.02 – 2.88 (m, 1H), 2.45 – 2.22 (m, 1H), 1.78 – 1.57 (m, 1H). HRMS (ESI): *m/z* calcd C_18_H_16_FN_2_O_2_ (M + H^+^) = 311.1196, found = 311.1193.

4-(4-Chlorophenyl)-8-nitro-3a,4,5,9b-tetrahydro-3*H*-cyclopenta[*c*]quinoline (37)

Pale brown solid, yield: 64%. ^1^H NMR (300 MHz, DMSO-*d*_6_) δ 7.95 (d, *J* = 2.7 Hz, 1H), 7.86 (dd, *J* = 9.0, 2.7 Hz, 1H), 7.50 (s, 4H), 7.29 (s, 1H), 6.85 (d, *J* = 9.1 Hz, 1H), 6.10 – 6.01 (m, 1H), 5.65 (d, *J* = 5.6 Hz, 1H), 4.80 (d, *J* = 3.6 Hz, 1H), 4.13 (d, *J* = 8.5 Hz, 1H), 2.95 (dd, *J* = 10.8, 7.5 Hz, 1H), 2.44 – 2.21 (m, 1H), 1.74 – 1.60 (m, 1H). HRMS (ESI): *m/z* calcd C_18_H_16_ClN_2_O_2_ (M + H^+^) = 327.0900, found = 327.0903.

4-(4-Bromophenyl)-8-nitro-3a,4,5,9b-tetrahydro-3*H*-cyclopenta[*c*]quinoline (38)

Pale brown solid, yield: 84%. ^1^H NMR (300 MHz, DMSO-*d*_6_) δ 7.94 (d, *J* = 2.7 Hz, 1H), 7.86 (dd, *J* = 9.0, 2.7 Hz, 1H), 7.67 – 7.58 (m, 2H), 7.48 – 7.39 (m, 2H), 7.28 (s, 1H), 6.85 (d, *J* = 9.0 Hz, 1H), 6.05 (td, *J* = 3.4, 1.7 Hz, 1H), 5.72 – 5.57 (m, 1H), 4.78 (d, *J* = 3.5 Hz, 1H), 4.12 (d, *J* = 8.7 Hz, 1H), 2.96 (d, *J* = 9.5 Hz, 1H), 2.43 – 2.21 (m, 1H), 1.66 (dd, *J* = 15.4, 8.5 Hz, 1H). HRMS (ESI): *m/z* calcd C_18_H_16_BrN_2_O_2_ (M + H^+^) = 371.0395, found = 371.0391.

4-(4-Iodophenyl)-8-nitro-3a,4,5,9b-tetrahydro-3*H*-cyclopenta[*c*]quinoline (39)

Pale brown solid, yield: 88%. ^1^H NMR (300 MHz, DMSO-*d*_6_) δ 7.94 (d, *J* = 2.7 Hz, 1H), 7.86 (dd, *J* = 9.0, 2.7 Hz, 1H), 7.79 (d, *J* = 8.4 Hz, 2H), 7.29 (d, *J* = 8.3 Hz, 3H), 6.85 (d, *J* = 9.0 Hz, 1H), 6.09 – 6.00 (m, 1H), 5.68 – 5.60 (m, 1H), 4.76 (d, *J* = 3.6 Hz, 1H), 4.12 (d, *J* = 8.6 Hz, 1H), 3.00 – 2.88 (m, 1H), 2.38 – 2.23 (m, 1H), 1.74 – 1.60 (m, 1H). HRMS (ESI): *m/z* calcd C_18_H_16_IN_2_O_2_ (M + H^+^) = 419.0256, found = 419.0260.

8-Nitro-4-(4-nitrophenyl)-3a,4,5,9b-tetrahydro-3*H*-cyclopenta[*c*]quinoline (40)

Yellow solid, yield: 90%. ^1^H NMR (300 MHz, DMSO-*d*_6_) δ 8.37 – 8.26 (m, 2H), 7.98 (d, *J* = 2.6 Hz, 1H), 7.89 (dd, *J* = 9.0, 2.6 Hz, 1H), 7.77 (d, *J* = 8.7 Hz, 2H), 7.40 (s, 1H), 6.88 (d, *J* = 9.1 Hz, 1H), 6.07 (dd, *J* = 4.6, 2.3 Hz, 1H), 5.65 (d, *J* = 5.7 Hz, 1H), 4.97 (d, *J* = 3.6 Hz, 1H), 4.17 (d, *J* = 8.4 Hz, 1H), 3.14 – 2.95 (m, 1H), 2.41 – 2.20 (m, 1H), 1.63 (dd, *J* = 15.8, 8.5 Hz, 1H). HRMS (ESI): *m/z* calcd C_18_H_16_N_3_O_4_ (M + H^+^) = 338.1141, found = 338.1140.

4-(8-Nitro-3a,4,5,9b-tetrahydro-3*H*-cyclopenta[*c*]quinolin-4-yl)benzonitrile (41)

Yellow solid, yield: 90%. ^1^H NMR (300 MHz, DMSO-*d*_6_) δ 8.01 – 7.82 (m, 4H), 7.68 (d, *J* = 8.0 Hz, 2H), 7.34 (s, 1H), 6.86 (d, *J* = 9.0 Hz, 1H), 6.10 – 6.02 (m, 1H), 5.67 – 5.59 (m, 1H), 4.90 (d, *J* = 3.6 Hz, 1H), 4.14 (d, *J* = 8.5 Hz, 1H), 3.05 – 2.96 (m, 1H), 2.36 – 2.21 (m, 1H), 1.63 (dt, *J* = 15.6, 5.4 Hz, 1H). HRMS (ESI): *m/z* calcd C_19_H_16_N_3_O_2_ (M + H^+^) = 318.1243, found = 318.1236.

4-(8-Nitro-3a,4,5,9b-tetrahydro-3*H*-cyclopenta[*c*]quinolin-4-yl)phenol (42, iBAP-II)

Yellow solid, yield: 50%. ^1^H NMR (300 MHz, DMSO-*d*_6_) δ 9.43 (s, 1H), 7.93 (d, *J* = 2.6 Hz, 1H), 7.84 (dd, *J* = 9.0, 2.7 Hz, 1H), 7.25 (d, *J* = 8.4 Hz, 3H), 6.83 (dd, *J* = 8.6, 5.4 Hz, 3H), 6.05 (dt, *J* = 5.7, 2.6 Hz, 1H), 5.66 (d, *J* = 5.6 Hz, 1H), 4.68 (d, *J* = 3.5 Hz, 1H), 4.09 (d, *J* = 8.6 Hz, 1H), 2.89 (dtd, *J* = 11.7, 9.0, 3.1 Hz, 1H), 2.46 – 2.27 (m, 1H), 1.72 (ddd, *J* = 16.9, 8.3, 2.4 Hz, 1H). HRMS (ESI): *m/z* calcd C_18_H_17_N_2_O_3_ (M + H^+^) = 309.1239, found = 309.1238.

8-Nitro-4-(p-tolyl)-3a,4,5,9b-tetrahydro-3*H*-cyclopenta[*c*]quinoline (43)

Yellow solid, yield: 43%. ^1^H NMR (300 MHz, DMSO-*d*_6_) δ 7.93 (d, *J* = 2.7 Hz, 1H), 7.84 (dd, *J* = 9.0, 2.7 Hz, 1H), 7.34 (d, *J* = 7.7 Hz, 2H), 7.28 – 7.19 (m, 3H), 6.85 (d, *J* = 9.0 Hz, 1H), 6.08 – 5.99 (m, 1H), 5.64 (d, *J* = 5.7 Hz, 1H), 4.74 (d, *J* = 3.6 Hz, 1H), 4.11 (d, *J* = 8.6 Hz, 1H), 2.98 – 2.89 (m, 1H), 2.45 – 2.24 (m, 4H), 1.67 (dd, *J* = 16.4, 8.4 Hz, 1H). HRMS (ESI): *m/z* calcd C_19_H_19_N_2_O_2_ (M + H^+^) = 307.1447, found = 307.1442.

4-(4-Methoxyphenyl)-8-nitro-3a,4,5,9b-tetrahydro-3*H*-cyclopenta[*c*]quinoline (44)

Yellow solid, yield: 39%. ^1^H NMR (300 MHz, DMSO-*d*_6_) δ 7.93 (d, *J* = 2.3 Hz, 1H), 7.84 (dd, *J* = 9.0, 2.7 Hz, 1H), 7.37 (d, *J* = 8.7 Hz, 2H), 7.24 (s, 1H), 6.98 (d, *J* = 8.7 Hz, 2H), 6.84 (d, *J* = 9.0 Hz, 1H), 6.09 – 5.99 (m, 1H), 5.64 (d, *J* = 5.7 Hz, 1H), 4.72 (d, *J* = 3.5 Hz, 1H), 4.15 – 4.05 (m, 1H), 3.79 (s, 3H), 2.97 – 2.83 (m, 1H), 2.43 – 2.28 (m, 1H), 1.76 – 1.62 (m, 1H). HRMS (ESI): *m/z* calcd C_19_H_19_N_2_O_3_ (M + H^+^) = 323.1396, found = 323.1400.

4-(4-Butoxyphenyl)-8-nitro-3a,4,5,9b-tetrahydro-3*H*-cyclopenta[*c*]quinoline (45)

Pale brown solid, yield: 35%. ^1^H NMR (300 MHz, DMSO-*d*_6_) δ 7.99 – 7.80 (m, 2H), 7.52 – 7.21 (m, 3H), 6.98 (d, *J* = 8.7 Hz, 2H), 6.85 (d, *J* =9.0 Hz, 1H), 6.12 – 5.93 (m, 1H), 5.65 (d, *J* = 5.7 Hz, 1H), 4.73 (d, *J* = 3.5 Hz, 1H), 4.18 – 3.91 (m, 3H), 2.92 (d, *J* = 9.5 Hz, 1H), 2.45 – 2.27 (m, 1H), 1.81 – 1.63 (m, 3H), 1.55 – 1.39 (m, 2H), 0.97 (t, *J* = 7.3 Hz, 3H). HRMS (ESI): *m/z* calcd C_22_H_25_N_2_O_3_ (M + H^+^) = 365.1865, found = 365.1856.

4-(4-(Tert-butyl)phenyl)-8-nitro-3a,4,5,9b-tetrahydro-3*H*-cyclopenta[*c*]quinoline (46)

Yellow solid, yield: 45%. ^1^H NMR (300 MHz, DMSO-*d*_6_) δ 7.94 (d, *J* = 2.6 Hz, 1H), 7.85 (dd, *J* = 9.0, 2.6 Hz, 1H), 7.50 – 7.35 (m, 4H), 7.29 (s, 1H), 6.83 (d, *J* = 9.1 Hz, 1H), 6.05 (dt, *J* = 5.9, 2.5 Hz, 1H), 5.66 (d, *J* = 5.6 Hz, 1H), 4.75 (d, *J* = 3.5 Hz, 1H), 4.13 (d, *J* = 8.6 Hz, 1H), 3.00 – 2.90 (m, 1H), 2.48 – 2.34 (m, 1H), 1.79 – 1.65 (m, 1H), 1.32 (s, 9H). HRMS (ESI): *m/z* calcd C_22_H_25_N_2_O_3_ (M + H^+^) = 349.1916, found = 349.1914.

**Supplementary Figure Legend**

**Supplementary Figure 1.**

**Synthesis of 4-phenyl-3a,4,5,9b-tetrahydro-3*H*-cyclopenta[*c*]quinoline (iBAP) derivatives.**

A) The structure of iBAP. B) Chemical synthesis of iBAP analogs. Reagents and conditions: (a) cyclopentadiene, TFA, MeCN, 0 ^o^C. C) Alignment by CLUSTALW analysis shows the similarity of the catalytic domain between human BAP1 and Drosophila Calypso. D) The receptor-ligand interactions for the top-ranked binding poses of BAP inhibitors obtained after conducting the molecular docking studies on Calypso (PDB: 6HGC) using the CDOCKER algorithm in Discovery Studio 4.5. E) Receptor-ligand interactions of molecular docking for iBAP and iBAP-II in Calypso’s catalytic site. F) Superposition of Calypso catalytic domain (dark blue) with catalytic domains of UCH-L1 (wheat), UCHL3 (orange), and UCH-L5 (light blue). Active site residues of Calypso (C131, H213 and D228) are compared with corresponding residues of other UCHs. G) The epoxy-activated agarose beads were incubated with either DMSO or iBAP-II in the presence of 50 mM NaOH for 6 hours, followed by blocking with 100 mM Glycine overnight. Then the beads were washed with PBS three times, followed by incubation with either recombinant his-tagged BAP1 protein or nuclear extract from NCI-H1963 cells for an additional 6 hours. Finally, the beads were washed by PBS for three times, and boiled in SDS loading buffer. The bound BAP1 protein were determined by either Coomassie blue staining (left panel) or western blot (right panel).

**Supplementary Figure 2.**

**Inhibition of BAP1 leads to ASXL3 degradation in small cell lung cancer cells.**

A) The relative protein levels of ASXL1-3, BAP1, HCFC1, FOXK1, and FOXK2 from Figure 2A were quantified by ImageJ. NCI-H1963 cells were treated with CHX (50 μg/ml) from different time points. Whole cell lysate was collected at each time point, and the total protein levels of ASXL1, ASXL2, ASXL3, and BAP1 were determined by western blot (B) and quantified by ImageJ (C). HSP90 was used as the internal control, n=2. D) NCI-H1963 cells were treated with iBAP (10, 20 μM) or iBAP-II (10, 20 μM) for 24 hours. RNA-seq was performed and cells treated with DMSO were used as the control, n=2. E) Pathway analysis was performed with metascape with the downregulated genes (LogFC > 0.5, p < 0.01, n=1442) and upregulated genes (LogFC > 0.5, p < 0.01, n=1752) in iBAP-II treated cells.

**Supplementary Figure 3.**

**BAP1 inactivation suppresses RING1B-dependent ASCL1 expression.**

A) The relative mRNA levels of ASCL1 were determined by real-time PCR in NCI-H748 and NCI-H1882 cells treated with either DMSO or iBAP-II (20 μM), n=3. Two-tailed unpaired Student’s t test. **P < 0.01; *P < 0.05. B) The protein levels of ASCL1 were determined by western blot in NCI-H748 and NCI-H1882 cells treated with either DMSO or iBAP-II (20 μM). HSP90 was used as the internal control, n=2. C) Track examples show the expression of *ASCL1* in cells treated with either DMSO, dBET6 (300 nM), JQ1 (1 μM), or IBET-151 (1 μM), n=2. D) Whole cell lysates were used for western blot with BAP1 antibody in human SCLC cell line NCI-H1963 transduced with either non-targeting CRISPR gRNA or BAP1-specific gRNA. HSP90 was used as an internal control, n=2. E) The RNA-seq data for the expression of the *ASCL1* gene in NCI-H1963 cells transduced with either non-targeting CRISPR gRNA or BAP1-specific gRNAs, n = 2, two-tailed unpaired Student’s t test. **P < 0.01; *P < 0.05. F) Representative track example that shows ASCL1 occupancy between cells treated with either DMSO or iBAP-II at *RNF183* and *DMPK* gene loci. G) Pathway analysis was performed with metascape with the genes that were downregulated by iBAP-II treatment and upregulated by RING1B depletion with two distinct shRNAs (n=739). H) The average plot shows the occupancy of BAP1 and RING1B centered on TSS at the 739 gene loci. I) The average plot shows the chromatin accessibility determined by ATAC-seq (GSE164247) centered on TSS at the 739 gene loci. J) The RNA-seq data for the expression of *ASCL1* gene in NCI-H1963 cells treated with either DMSO or EZH2 inhibitor GSK126 (2 μM) for four days, n = 2, two-tailed unpaired Student’s t test. NS = non-significant.

**Supplementary Figure 4.**

**BAP1 is a common essential factor for SCLC viability.**

A) Whole cell lysates were used for western blot with BAP1 antibody in mouse SCLC cell line KP1 transduced with either non-targeting CRISPR gRNA or BAP1-specific gRNAs. HSP90 was used as an internal control, n=2. B) Mouse SCLC cell line KP1 were transduced with either non-targeting CRISPR gRNA or BAP1-specific gRNAs for four days. The cell numbers were determined by cell counting assay, n=3. Two-tailed unpaired Student’s *t*-test. **P < 0.01; *P < 0.05. C) 5.0 × 10^5^ of BAP1-WT and -depleted cells were inoculated into the right flank of athymic nude mice (n=5 for each group). The tumor growth was determined every five days. A two-tailed unpaired Student's *t*-test was used for statistical analysis. **P < 0.01; *P < 0.05. D) A representative photograph of several tumor masses to visually show differences in the sizes between non-targeting CRISPR gRNA and BAP1-specific gRNAs. E) The protein levels of BAP1 in NCI-H1963, NCI-H748, NCI-H1882, KP3, and NCI-H226 cell lines were determined by western blot. HSP90 was used as an internal control, n=2. F) The BAP1-null SCLC cell line NCI-H226 was treated with various concentrations of iBAP-II for 72 hours. The cell number was determined by cell counting assay, n=3. G) The IC50 from Figure 4B and Figure S4F were calculated. H) The mRNA levels of the MYC pathway genes *SNRPG*, *MRPL23*, *HNRNPR*, *LSM7*, and *ODC1* were determined by real-time PCR in three different SCLC cells treated with either DMSO or iBAP-II, n=3. I) Gene Set Enrichment Analysis (GSEA) of SCLC gene signature enrichment in iBAP-II treatment condition. RNA-seq data of SCLC patient samples (n=79) and normal lung tissues (n=7) were obtained from GEO database (GSE60052). A total of 500 top downregulated genes in SCLC tumors versus normal tissues were selected based on the log2 fold change < -1.98; A total of 335 top upregulated genes in SCLC tumors versus normal tissues were selected based on the log2 fold change > 1.

**Supplementary Table 1**

| No. | R_1_ | R_2_ | MW | Structure |
| --- | --- | --- | --- | --- |
| PBA-1-1 | 4-H | 4-H | 247.1361 |  |
| PBA-1-2 | 2-CH3 | 4-H | 261.1517 |  |
| PBA-1-3 | 2-NO2 | 4-H | 292.1212 |  |
| PBA-1-4 | 3-NO2 | 4-H | 292.1212 |  |
| PBA-1-5 | 4-NO2 | 4-H | 292.1212 |  |
| PBA-1-6 | 3,4,5-tri-OCH3 | 4-H | 337.1678 |  |
| PBA-1-7 | 4-F | 4-H | 265.1267 |  |
| PBA-1-8 | 4-Cl | 4-H | 281.0971 |  |
| PBA-1-9 | 4-Br | 4-H | 325.0466 |  |
| PBA-1-10 | 4-I | 4-H | 373.0327 |  |
| PBA-1-11 | 4-CN | 4-H | 272.1313 |  |
| PBA-1-12 | 4-COOH | 4-H | 291.1259 |  |
| PBA-1-13 | 4-PhCH2O | 4-H | 353.1780 |  |
| PBA-1-14 | 4-OH | 4-H | 263.1310 |  |
| PBA-1-15 | 4-OCH3 | 4-H | 277.1467 |  |
| PBA-1-16 | 4-CH2CH3 | 4-H | 275.1674 |  |
| PBA-1-17 | 4-NO2 | 2-F | 310.1118 |  |
| PBA-1-18 | 4-NO2 | 2-Br | 370.0317 |  |
| PBA-1-19 | 4-NO2 | 2-I | 418.0178 |  |
| PBA-1-20 | 4-NO2 | 2-NO2 | 337.1063 |  |
| PBA-1-21 | 4-NO2 | 2-CN | 317.1164 |  |
| PBA-1-22 | 4-NO2 | 2-CF3 | 360.1086 |  |
| PBA-1-23 | 4-NO2 | 2,3-Di-Cl | 360.0432 |  |
| PBA-1-24 | 4-NO2 | 2-OCH3 | 322.1317 |  |
| PBA-1-25 | 4-NO2 | 3-F | 310.1118 |  |
| PBA-1-26 | 4-NO2 | 3-Cl | 326.0822 |  |
| PBA-1-27 | 4-NO2 | 3-Br | 370.0317 |  |
| PBA-1-28 | 4-NO2 | 3-I | 418.0178 |  |
| PBA-1-29 | 4-NO2 | 3-NO2 | 337.1063 |  |
| PBA-1-30 | 4-NO2 | 3-CN | 317.1164 |  |
| PBA-1-31 | 4-NO2 | 3-CF3 | 360.1086 |  |
| PBA-1-32 | 4-NO2 | 3-OH | 308.1161 |  |
| PBA-1-33 | 4-NO2 | 3-CH3 | 306.1368 |  |
| PBA-1-34 | 4-NO2 | 3-OCH3 | 322.1317 |  |
| PBA-1-35 | 4-NO2 | 3,4-di-och3 | 352.1423 |  |
| PBA-1-36 | 4-NO2 | 4-F | 310.1118 |  |
| PBA-1-37 | 4-NO2 | 4-Cl | 326.0822 |  |
| PBA-1-38 | 4-NO2 | 4-Br | 370.0317 |  |
| PBA-1-39 | 4-NO2 | 4-I | 418.0178 |  |
| PBA-1-40 | 4-NO2 | 4-NO2 | 337.1063 |  |
| PBA-1-41 | 4-NO2 | 4-CN | 317.1164 |  |
| PBA-1-42 | 4-NO2 | 4-OH | 308.1161 |  |
| PBA-1-43 | 4-NO2 | 4-CH3 | 306.1368 |  |
| PBA-1-44 | 4-NO2 | 4-OCH3 | 322.1317 |  |
| PBA-1-45 | 4-NO2 | 4- | 364.1787 |  |
| PBA-1-46 | 4-NO2 | 4-C(CH3)3 | 348.1838 |  |
