# Supplementary figures and images for: Therapeutic targeting of BAP1/ASXL3 sub-complex in ASCL1-dependent small cell lung cancer
Source: Oncogene. 2022 Feb 22;41(15):2152–62. doi: 10.1038/s41388-022-02240-x (PMC8993689; doi:10.1038/s41388-022-02240-x)

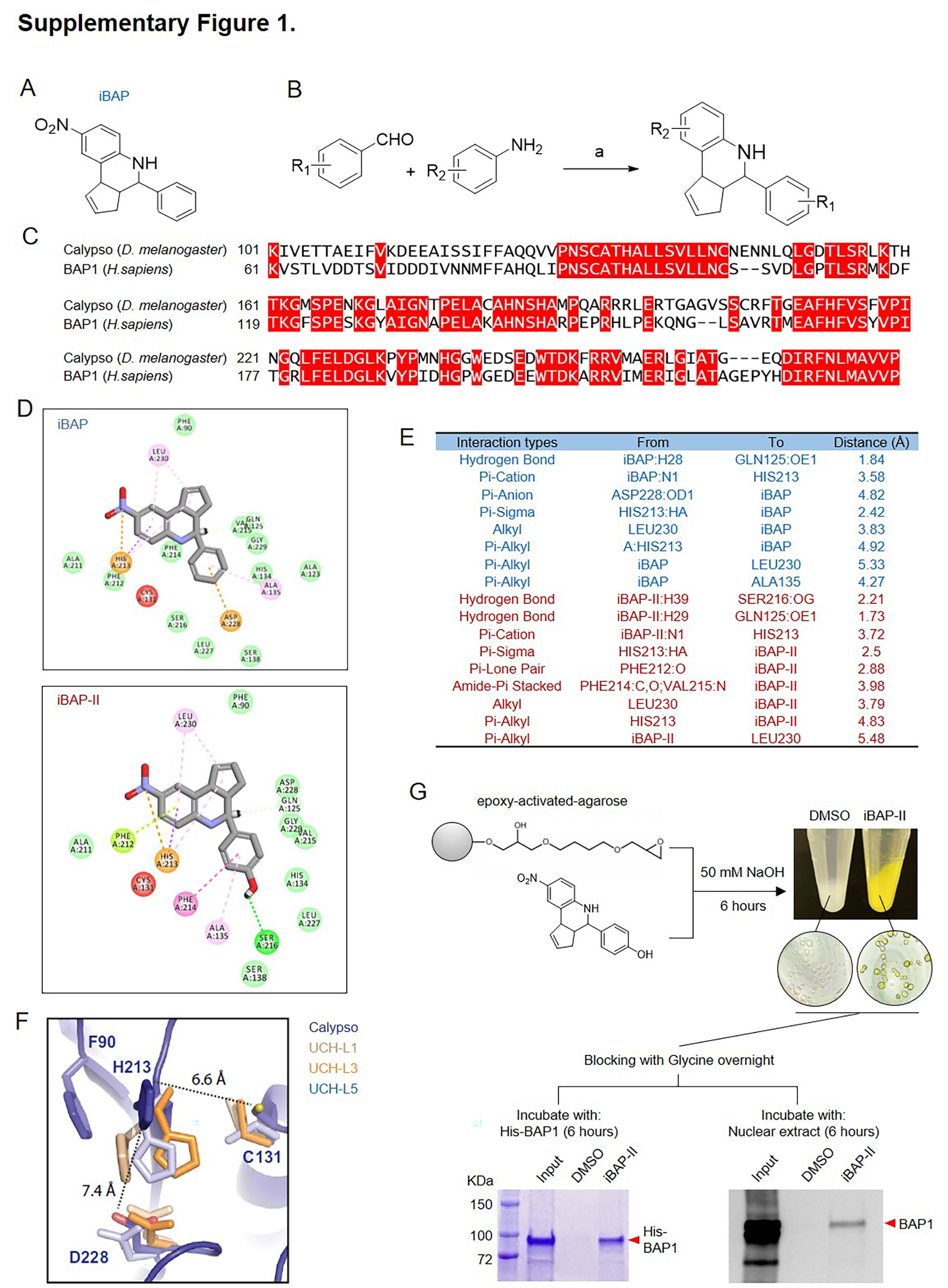

Supplement: Supplementary file 2 — Supplementary Figure 1 [file 41388_2022_2240_MOESM2_ESM.jpg]

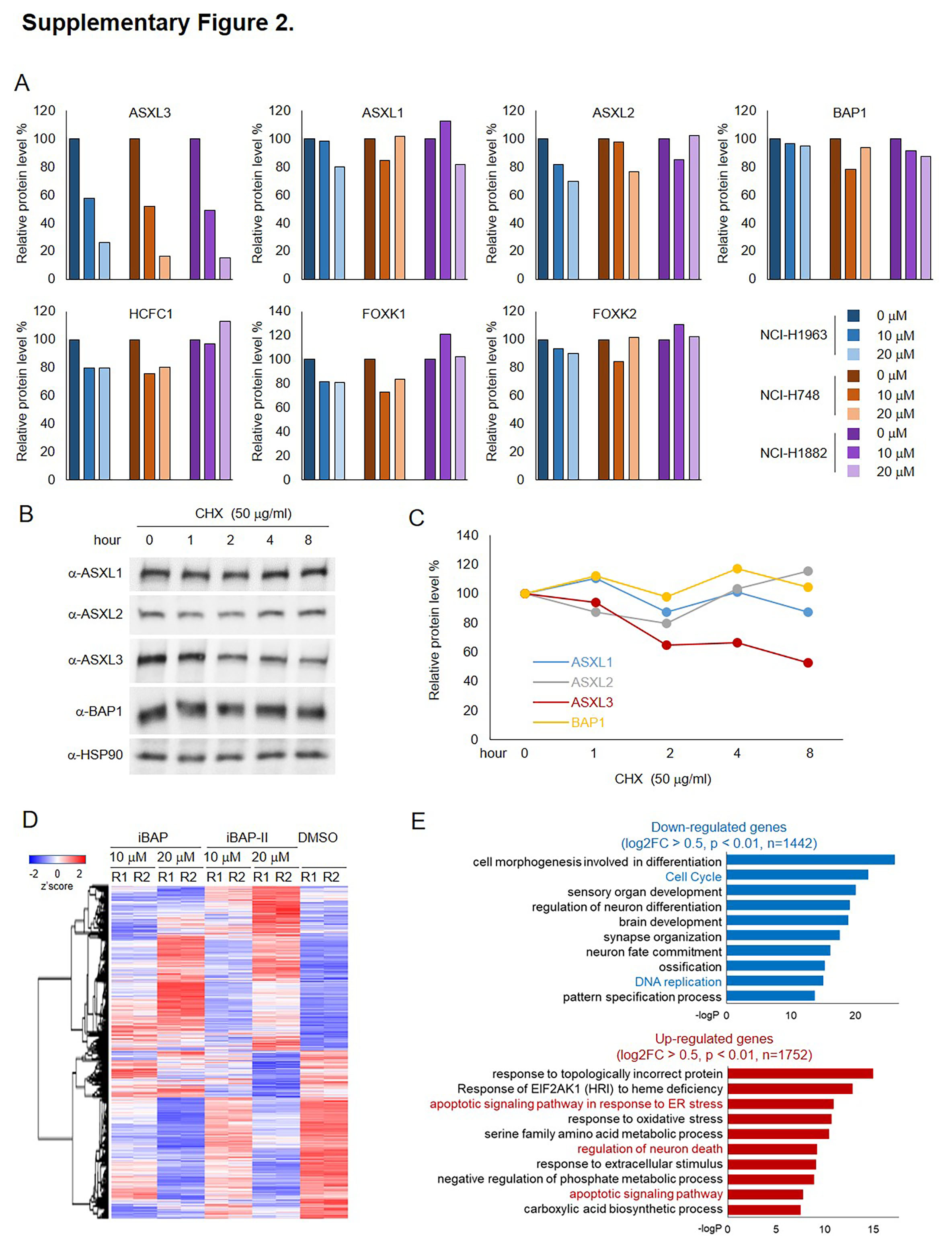

Supplement: Supplementary file 3 — Supplementary Figure 2 [file 41388_2022_2240_MOESM3_ESM.jpg]

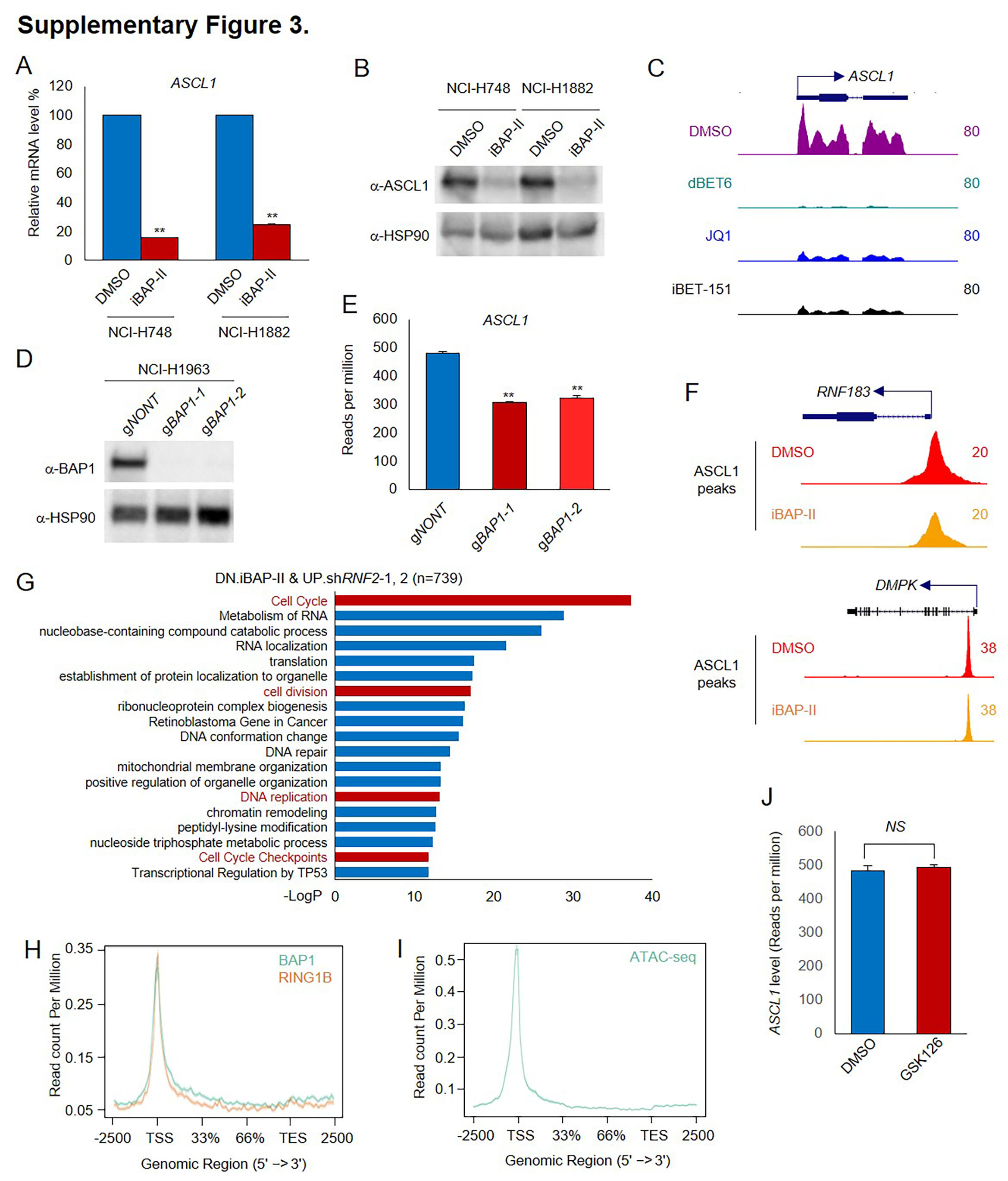

Supplement: Supplementary file 4 — Supplementary Figure 3 [file 41388_2022_2240_MOESM4_ESM.jpg]

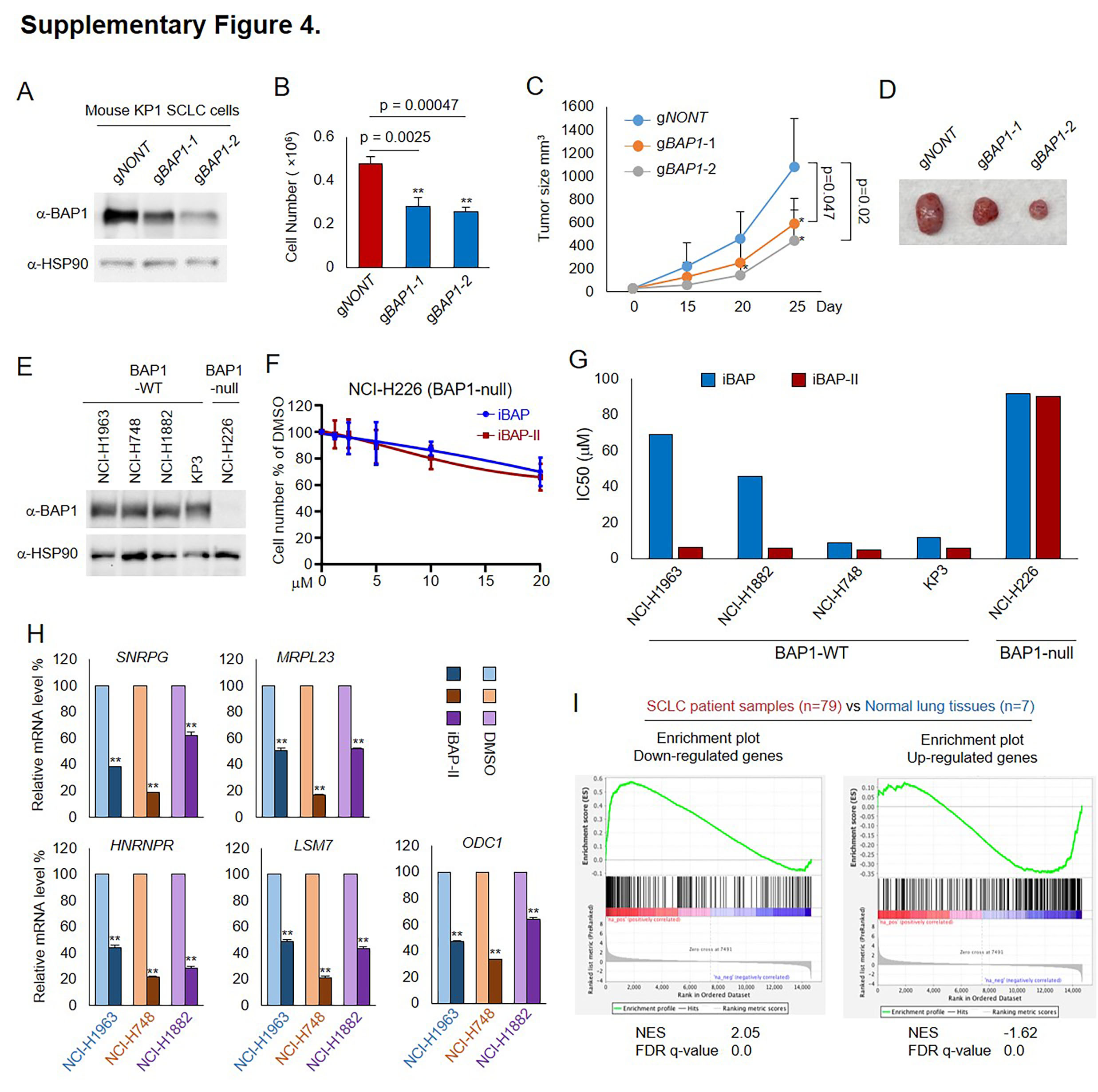

Supplement: Supplementary file 5 — Supplementary Figure 4 [file 41388_2022_2240_MOESM5_ESM.jpg]
